# How Secondary and Tertiary Amide Moieties are Molecular Stations for Dibenzo-24-crown-8 in [2]Rotaxane Molecular Shuttles?

**DOI:** 10.3390/molecules22112017

**Published:** 2017-11-21

**Authors:** Benjamin Riss-Yaw, Justine Morin, Caroline Clavel, Frédéric Coutrot

**Affiliations:** Supramolecular Machines and ARchitectures Team, Institut des Biomolécules Max Mousseron (IBMM) UMR 5247 CNRS, Université Montpellier, ENSCM, Case Courrier 1706, Bâtiment Chimie (17), 3ème étage, Faculté des Sciences, Place Eugène Bataillon, 34095 Montpellier CEDEX 5, France; benjamin.riss-yaw@etu.umontpellier.fr (B.R.-Y.); justine.morin@etu.umontpellier.fr (J.M.); caroline.clavel@umontpellier.fr (C.C.)

**Keywords:** molecular machine, rotaxane, amide, ammonium, amine, dibenzo-24-crown-8, template

## Abstract

Interlocked molecular machines like [2]rotaxanes are intriguing aesthetic molecules. The control of the localization of the macrocycle, which surrounds a molecular axle, along the thread leads to translational isomers of very different properties. Although many moieties have been used as sites of interactions for crown ethers, the very straightforwardly obtained amide motif has more rarely been envisaged as molecular station. In this article, we report the use of secondary and tertiary amide moieties as efficient secondary molecular station in pH-sensitive molecular shuttles. Depending on the *N*-substitution of the amide station, and on deprotonation or deprotonation-carbamoylation, the actuation of the molecular machinery differs accordingly to very distinct interactions between the axle and the DB24C8.

## 1. Introduction

Since the first interlocked molecular shuttles were reported in the literature in 1994 [1,2], several new systems of recognition have been used to control the localization of a macrocycle around either another macrocycle or a thread in more or less sophisticated interlocked molecules. With the dibenzo-24-crown-8 macrocycle (DB24C8), the use of ammonium [3], as template to direct the interlocking process, is probably the most encountered. This template also presents the advantage of being possibly concealed through deprotonation, so that the interactions between the template and the macrocycle become annihilated. This possibility is of interest for the construction of multi-stable molecular shuttles, especially if another thread’s moiety of initial weaker affinity is present in the thread. In this case, the macrocycle can shuttle along the thread and sit around the second station. The process can be reversible through protonation. Hence, reporting new stations of weaker affinity for the macrocycle might be of interest. Several secondary stations for the DB24C8 have been reported in the literature to date. As a non-comprehensive list of them, we can cite the following positively charged stations: bipyridinium [4,5,6,7,8,9,10], 1,2-bis-(pyridinium)ethane [11,12,13,14,15,16,17,18,19,20,21], triazolium [22,23,24,25,26,27,28,29,30], pyridinium [31,32,33], pyridinium amide [34,35,36,37], imidazolium [38,39,40,41] moieties, oxidized ferrocene [42], etc. Some neutral molecular stations are also important to be mentioned, such as urea [43], thiourea [44], carbamate [45,46,47,48,49,50], isoxazole [51], and benzylic ester [52], that usually interact either through π-π stacking with the aromatic rings of the crown ether, or through hydrogen bonding with the oxygen atoms of the crown ether. All of these sites of interactions are weaker stations than the ammonium moiety. However, they are better site of recognitions than the amine moiety that directly derives from deprotonation of the best ammonium station, allowing the pH-sensitive actuation of the molecular machinery. To the best of our knowledge, although one can find in the literature some DB24C8-containing [2]rotaxanes that involve amide moieties [3,53,54,55], only a few studies have investigated the utilization of a single secondary amide moiety as an efficient secondary molecular station for the DB24C8 [56,57]. In this paper, we straightforwardly synthesized [2]rotaxane molecular shuttles that combine a strong ammonium station and either a secondary or a tertiary amide station. Depending on the class of the amide station, the molecular machinery actuates differently through the concealing/revealing of the ammonium moiety.

## 2. Results

### 2.1. Synthetic Access to Molecular Shuttles

**Scheme 1:**
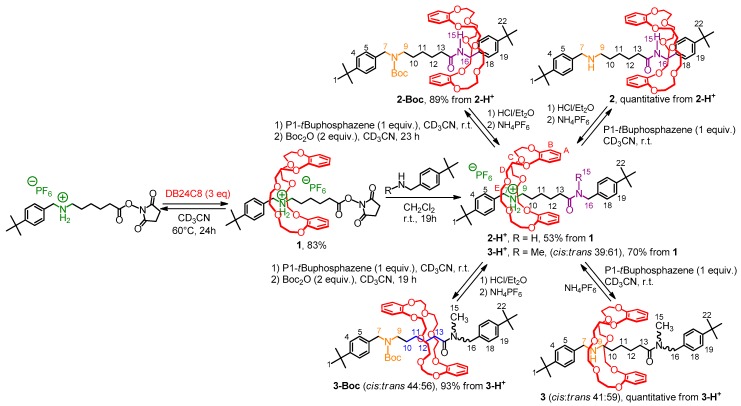
Synthesis of secondary and tertiary amide-containing [2]rotaxane molecular shuttles.

The [2]rotaxane molecular shuttles were obtained from the isolable active ester-containing [2]rotaxane building-block **1** (Scheme 1) [24]. A very efficient slippage process allowed the formation of the *N*-hydroxysuccinimide ester-containing [2]rotaxane **1** in very good yield. Once the rotaxane building-block **1** isolated, addition of either *tert*-butylbenzylamine or *N*-methyl-*tert*-butylbenzylamine provided directly the protonated secondary and tertiary amide-containing [2]rotaxanes **2-H^+^** and **3-H^+^**, respectively.

At the protonated state, the DB24C8 is localized around the best ammonium station whatever the secondary or tertiary class of the amide. Shuttling of the macrocycle along the thread was then considered upon concealing the ammonium station. Deprotonation and deprotonation-carbamoylation of the ammonium were carried out. The deprotonation appeared quite difficult with standard bases such as tertiary amines, sodium hydroxide or DBU, as we already encountered this problem anteriorly with encircled ammonium moieties in interlocked [2]rotaxane species when no sufficiently strong secondary molecular station was present close to the ammonium one [58]. The use of the strong P1-*t*Bu-tris(dimethyl)phosphazene Schwesinger’s base (1 equiv) [59] nevertheless allowed the complete deprotonation of the ammonium moiety in situ. Deprotonation-carbamoylation was also achieved and provided the pure carbamoylated [2]rotaxanes **2-Boc** and **3-Boc** in very good yields (89% and 93%, respectively, after chromatography).

### 2.2. Characterizations of the Molecular Shuttles

**Figure 1:**
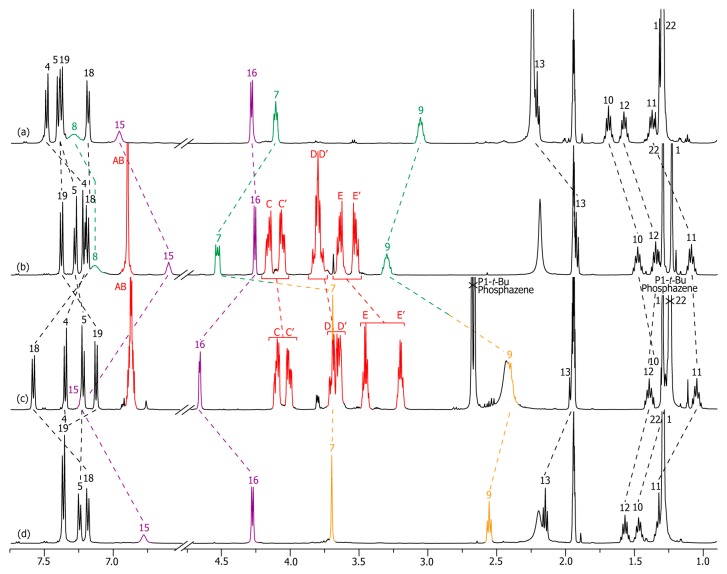
^1^H NMR (500 MHz, CD_3_CN, 298 K) comparison between the spectra of: (**a**) the protonated non-interlocked molecular axle **2u-H^+^**; (**b**) the protonated [2]rotaxane **2-H^+^**; (**c**) the deprotonated [2]rotaxane **2**; (**d**) the deprotonated non-interlocked analogue **2u**. The numbering and coloring of the signals correspond to those indicated in Scheme 1. All the ^1^H NMR spectra have been carried out at a concentration of 4.9 × 10^−2^ M.

In the secondary amide series, the localization of the DB24C8 at the protonated and deprotonated states ([2]rotaxanes **2-H^+^** and **2**) was demonstrated thanks to ^1^H NMR spectroscopy (Figure 1). The comparison between the ^1^H NMR spectrum of **2-H^+^** with that of the uncomplexed thread **2u-H^+^** highlights the presence and the localization of the DB24C8 around the best ammonium station (Figure 1a,b). Apart from the obvious appearance of H_A–E_ belonging to the DB24C8, hydrogen atoms H_7_ and H_9_ that are neighboring the ammonium moiety are both shifted downfield in **2-H^+^** (Δδ = 0.42 and 0.25 ppm, respectively) due to their hydrogen bonding interactions with the oxygen atoms of the crown ether. At the same time, hydrogen atoms H_10–13_, H_15_ and in a much lesser extent H_16_ are more or less shifted upfield in **2-H^+^** (Δδ = −0.22, −0.29, −0.23, −0.28, −0.36 and −0.03 ppm, respectively) because they experience the shielding region of the aromatic rings of the DB24C8.

Deprotonation of the ammonium triggered the shuttling of the DB24C8 around the secondary amide station (Scheme 1). More precisely, at the deprotonated state of the molecular shuttle, the DB24C8 interacts through hydrogen bonds with the amide hydrogen atom H_15_ and the next hydrogen atoms H_16_ (Figure 1b,c). Indeed, with respect to **2-H^+^**, in **2**, the hydrogen atoms H_7_, H_9_, and in a lesser extent H_10_ are obviously shifted upfield (Δδ = −0.91, −0.89, and −0.21 ppm, respectively) due to the deprotonation of the ammonium. Meanwhile, hydrogen atoms H_15_ and H_16_ are importantly shifted downfield (Δδ = +0.64 and +0.40 ppm, respectively). Concerning the methylenic hydrogen atoms H_E–E’_ of the DB24C8, they are shielded in **2** (Δδ = −0.19 and −0.32 ppm), because of their localization in the shielding region of the aromatic ring of the *N*-benzylamide extremity of the axle. One might also compare the localization of these hydrogen atoms H_E–E’_ in the two *co*-conformational states **2-H^+^** and **2**. By comparison with the free DB24C8, H_E–E’_ of each *co*-conformational state are shielded in the rotaxane architectures. However, the fact that the DB24C8 interacts with three centers (H_7–9_) neighboring the aromatic “left” end of the axle at the protonated state **2-H^+^** rather than only two centers (H_15–16_) next to the aromatic “right” end of the axle in the deprotonated state **2** induces a slight lower shielding of the H_E–E’_ signals in **2-H^+^**.

The accurate localization of the DB24C8 that interacts through hydrogen bonding with only atoms H_15_ and H_16_ in **2** is corroborated by the direct comparison between the ^1^H NMR spectra of rotaxane **2** and its uncomplexed analogue **2u** (Figure 1c,d). The same trend in chemical shift displacements are observed, i.e., H_15–16_ are the only deshielded hydrogen atoms in the interlocked architecture **2** (Δδ = +0.46 and +0.39 ppm). It is noteworthy that hydrogen atoms H_13_, which we imagine they could have interacted through hydrogen bonds with the oxygen atoms of the DB24C8, if one considered the acidity of hydrogen atoms next to an amide carbonyl, are on contrary shielded in the rotaxane **2** (Δδ = −0.20 ppm), like H_9–12_ are too (Δδ = −0.14, −0.21, −0.27, and −0.18 ppm, respectively).

**Figure 2:**
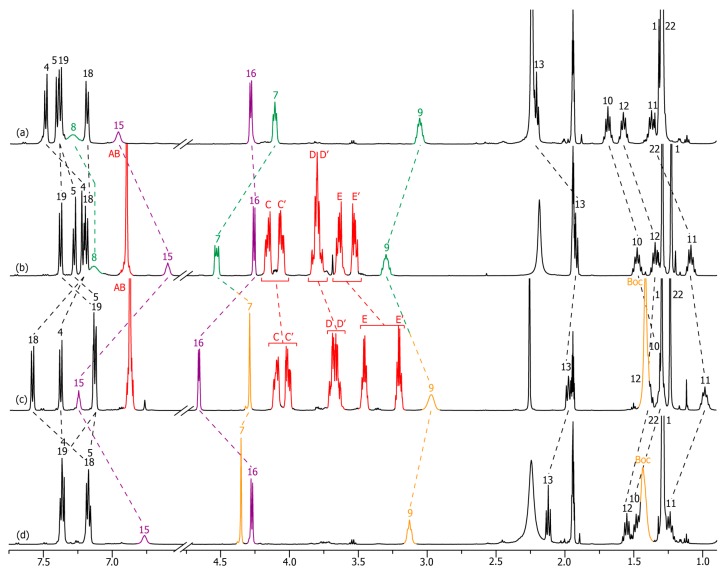
^1^H NMR (500 MHz, CD_3_CN, 298 K) comparison between the spectra of: (**a**) the protonated non-interlocked molecular axle **2u-H^+^**; (**b**) the protonated [2]rotaxane **2-H^+^**; (**c**) the carbamoylated [2]rotaxane **2-Boc**; (**d**) the carbamoylated non-interlocked analogue **2u-Boc**. The numbering and coloring of the signals correspond to those indicated in Scheme 1. All the ^1^H NMR spectra have been carried out at a concentration of 4.9 × 10^−2^ M.

Interestingly, and corroborating the fact that the ammonium moiety was very difficult to deprotonate, [36,60] removal of the P1-*t*Bu-tris(dimethyl)phosphazene from the in situ deprotonated rotaxane crude **2** unsuccessfully led, through silica or size-exclusion chromatographic column, to the protonated rotaxane **2-H^+^**. Deprotonation-carbamoylation of rotaxane **2-H^+^** was therefore achieved in order to obtain a pure translational *co*-conformer analogue. Hence, the isolated carbamoylated rotaxane **2-Boc** was easily obtained and isolated in a two-step sequence with an 89% overall yield from **2-H^+^** (Scheme 1). In the [2]rotaxane **2-Boc**, the localization of the DB24C8 along the thread was fully similar to that observed in **2**, highlighting the fact that the presence of the bulky Boc moiety had no influence on the localization of the macrocycle around the secondary amide station. Figure 2 shows the comparison of ^1^H NMR spectra that are necessary to demonstrate the accurate localization of the macrocycle along the thread. Apart from a difference of chemical shifts for H_7_ and H_9_ that is due to the carbamoylation of the next amine moiety, exactly the same explanations (downfield shifts due to hydrogen bonds between axle and macrocycle and shielding effect due to aromatic rings of both the DB24C8 and the axle’s extremities) can be stated for the system **2-H^+^**/**2-Boc** than those mentioned above for **2-H^+^**/**2**.

Tertiary amide motif was then envisaged in order to evaluate its propensity to act as a secondary molecular station for the DB24C8. To the best of our knowledge, no tertiary amide has been reported as a molecular station for crown ether to date, although one might benefit from the expected weakest affinity of the station for the macrocycle. Since the hydrogen atom, which is linked to the nitrogen atom of a primary or secondary amide, is replaced by a methyl substituent in the tertiary amide-containing rotaxanes **3** and **3-Boc**, we wondered how the DB24C8 would interact with the tertiary amide motif, and if it still does, which hydrogen atoms would be involved in the template site. Remarkably, changing from the secondary to the tertiary class of the amide induced a singular behavior of the macrocycle along the thread, and the localization of the DB24C8 and its interactions with the encircled axle were quite different after deprotonation or deprotonation-carbamoylation of the ammonium template.

**Figure 3:**
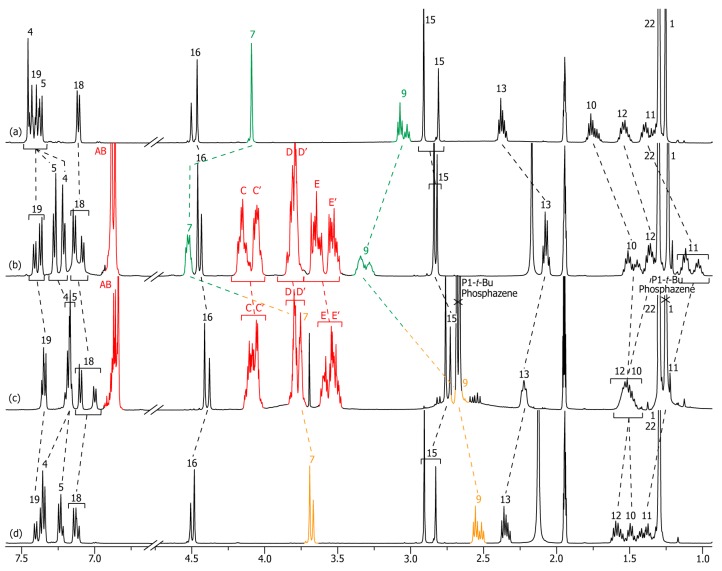
^1^H NMR (500 MHz, CD_3_CN, 298 K) comparison between the spectra of: (**a**) the protonated non-interlocked molecular axle **3u-H^+^**; (**b**) the protonated [2]rotaxane **3-H^+^**; (**c**) the deprotonated [2]rotaxane **3**; (**d**) the deprotonated non-interlocked analogue **3u**. The numbering and coloring of the signals correspond to those indicated in Scheme 1. All the ^1^H NMR spectra have been carried out at a concentration of 4.9 × 10^−2^ M.

In the protonated rotaxane **3-H^+^**, as for **2-H^+^**, the DB24C8 resides around the best ammonium station, which appears consistent with the fact that a tertiary amide was expected to be a worse molecular station than the secondary molecular station (Scheme 1, Figure 3). Hence, the same trend of chemical shift variations was noticed when comparing **3-H^+^** with its uncomplexed analogue **3u-H^+^** than that observed through the comparison between the secondary amide-containing rotaxane **2-H^+^** and its uncomplexed analogue **2u-H^+^** (Figure 1a,b and Figure 3a,b). Only one difference concerns the higher complexity of the ^1^H NMR spectra for the tertiary amide-containing compounds **3-H^+^**, **3**, **3-Boc**, and their respective non-interlocked analogues because of the *cis*-*trans* isomerism of the amide bond. Briefly, hydrogen atoms H_7_ and H_9_ are shifted downfield in the interlocked architecture (Δδ = +0.43 and +0.27 ppm, respectively), due to their implication in hydrogen-bonding with the oxygen atoms of the crown ether, while almost all the other hydrogen atoms are more or less shifted upfield because they experience the shielding effect of the aromatic ring of the DB24C8. Only the hydrogen atoms H_18–19_ and H_22_ do not undergo any variation of their chemical shifts, which corroborates the localization of the DB24C8 at the other side of the encircled axle, i.e., around the ammonium site.

Deprotonation of rotaxane **3-H^+^** using the P1-*t*Bu-tris(dimethyl)phosphazene resulted in no real change of the main localization of the macrocycle, albeit an obvious weaker interaction between the interlocked components was noticed. This result implies that the tertiary amide is a weaker molecular station than the amine moiety. The full deprotonation of rotaxane **3-H^+^** and the *co*-conformational state of the deprotonated rotaxane **3** are highlighted by the ^1^H NMR comparison between spectra of **3-H^+^** and **3** (Figure 3b,c). In particular, hydrogen atoms H_7_ and H_9_ are highly shifted upfield in **3** (Δδ = −0.77 and −0.65 ppm, respectively), but not as they should be if they were not interacting with the DB24C8 anymore (see below the comparison between the ^1^H NMR spectra of **3** and **3u**). Meanwhile, hydrogen atoms H_11–13_ are deshielded in **3** (Δδ = +0.19, +0.17, and +0.15 ppm, respectively), although the deprotonation of the ammonium moiety provides an amine moiety, which is a lesser electron-withdrawing group. One might therefore suggest that these hydrogen atoms experience a lower shielding effect of the DB24C8 than in **3-H^+^**, probably due to the weaker interactions between the amine moiety and the DB24C8 that allow a higher translational degree of freedom of this latter along the thread. This matches with the fact that hydrogen atoms H_15_ and H_16_ (right side of the axle) are slightly shielded in **3** with respect to **3-H^+^** (Δδ = −0.09 and −0.06 ppm, respectively), which might indicate that they undergo a slight shielding from the aromatic rings of the DB24C8. In fact, the accurate localization of the DB24C8 around the amine in **3** appears slightly different than that observed around the ammonium in rotaxane **3-H^+^**. The direct comparison between the ^1^H NMR spectra of **3** with that of its uncomplexed analogue **3u** reveals a site of weak hydrogen bonding interactions between the two interlocked components at the amine site (Figure 3c,d). Indeed, one can notice the slight downfield shift of hydrogen atoms H_7_ and H_9_ in **3** (Δδ = +0.05 ppm and +0.13 ppm, respectively). Although the macrocycle resides over the same region of the axle in **3** and **3-H^+^** (over the amine or the ammonium region, respectively), the downfield shifts of H_7_ and H_9_ observed in **3** (with respect to **3u**) are inferior than those observed in the protonated rotaxane **3-H^+^** (with respect to **3u-H^+^**). This is attributed to the weaker hydrogen bonds of the neutral amine station for the DB24C8 with respect to the positively charged ammonium station. More interestingly, the higher downfield shift of H_9_ than H_7_ (+0.13 vs. +0.05) in **3** (with respect to **3u**) compared to the higher downfield shift of H_7_ than H_9_ (+0.43 vs. +0.27) in **3-H^+^** (with respect to **3u-H^+^**) indicates a small change in the preorganization between the DB24C8 and the axle (the DB24C8 sits more over H_9_ than H_7_), which corroborates the higher shielding of the “right side” of the axle in **3**. At the exception of H_22_, all the other hydrogen atoms of the encircled thread are experiencing a slight shielding effect of the aromatic rings of the DB24C8. Besides, the methylenic hydrogens H_C–E_ of the DB24C8 are less split in the deprotonated rotaxane **3** than in **3-H^+^**. This observation corroborates the weaker hydrogen bonds involving the oxygen atoms of the DB24C8 and the amine station since strong hydrogen bonds are known to lower the rotation of σ bonds due to more constrained structure, hence to increase the splitting of the signals due to the better differentiation of geminal hydrogen atoms [36,37].

**Figure 4:**
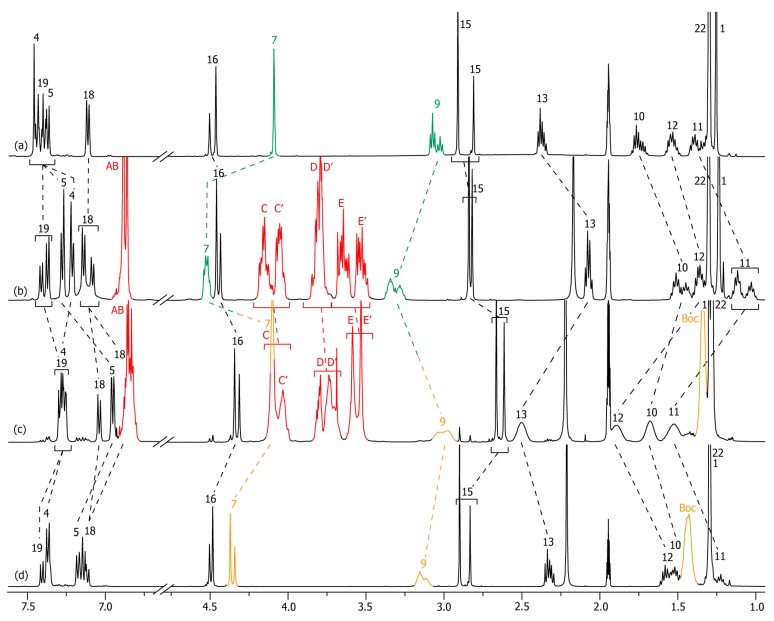
^1^H NMR (500 MHz, CD_3_CN, 298 K) comparison between the spectra of: (**a**) the protonated non-interlocked molecular axle **3u-H^+^**; (**b**) the protonated [2]rotaxane **3-H^+^**; (**c**) the carbamoylated [2]rotaxane **3-Boc**; (**d**) the carbamoylated non-interlocked analogue **3u-Boc**. The numbering and coloring of the signals correspond to those indicated in Scheme 1. All the ^1^H NMR spectra have been carried out at a concentration of 4.9 × 10^−2^ M.

Surprisingly, deprotonation-carbamoylation of **3-H^+^** resulted in a distinct *co*-conformational state, with respect to what occurred after the single deprotonation of **3-H^+^** (Scheme 1, Figure 4). Indeed, in the [2]rotaxane **3-Boc**, the DB24C8 interacts with the methylenic hydrogen atoms H_10–13_ through hydrogen bonding (Figure 4c,d). By comparing the ^1^H NMR spectra of the carbamoylated [2]rotaxane **3-Boc** and its uncomplexed thread **3u-Boc**, H_10–13_ are shifted downfield in the rotaxane architecture (Δδ = +0.15 ppm, +0.27 ppm, +0.21 ppm, and +0.18 ppm, respectively). At the same time, hydrogen atoms H_16_ (which participate through H-bond with the oxygen atoms of the DB24C8 in the secondary amide-containing [2]rotaxane **2**) are now shielded in **3-Boc** (Δδ = +0.16 ppm), probably because the oxygen atoms of the DB24C8 cannot interact with these hydrogen atoms due to the steric problem generated by the amide isomerism. Likewise, hydrogen atoms H_15_, H_7_, H_Boc_ and H_9_ are shielded in **3-Boc** (Δδ = −0.23, −0.25, −0.10, and −0.13 ppm, respectively) because they experience the shielding effect of the aromatic rings of the DB24C8.

**Figure 5:**
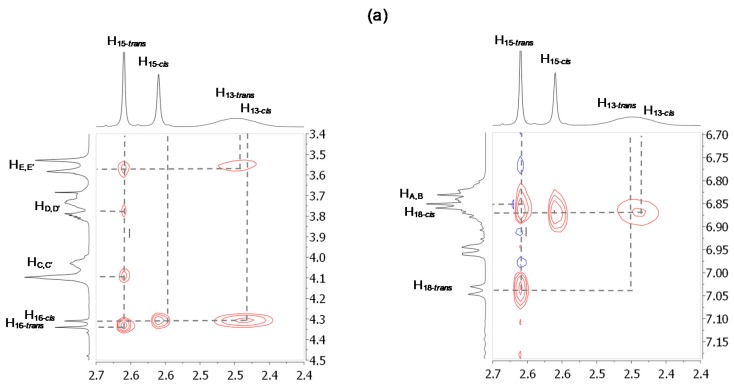

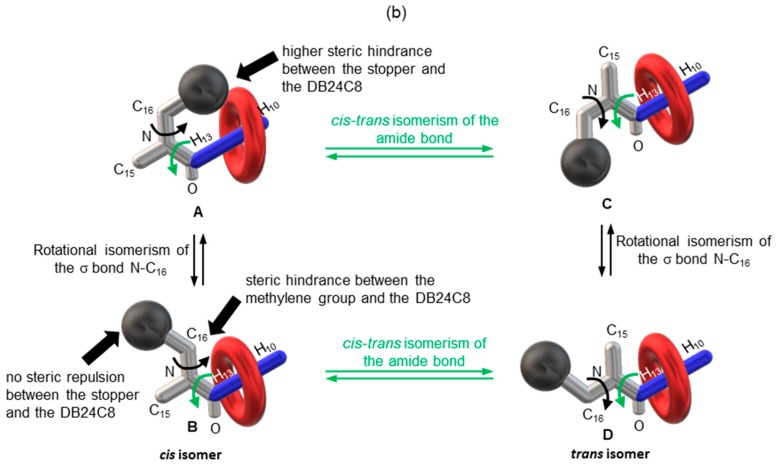
(**a**) Selected portions of 2D-ROESY ^1^H NMR spectrum (500 MHz, CD_3_CN, 298 K) of rotaxane **3-Boc**; (**b**) Cartoon representation highlighting the translational motion of the DB24C8 from H_10_ to H_13_ due to bonds rotation.

It is of particular interest to notice that the expected more acidic hydrogen atoms (i.e., H_13_) are not the most affected by the hydrogen bonds occurring with the oxygen atoms of the DB24C8. Instead, the next hydrogen atoms H_11_ and H_12_ are. One might suggest that both the *cis*-*trans* isomerism of the next tertiary amide and the rotation of the N-C_16_ σ bond, which occur quickly at room temperature [61], allow for the presence of isomers in which steric repulsions prevent the DB24C8 from interacting optimally with the most acidic hydrogens H_13_ (Figure 5b). The DB24C8 is therefore sterically forced to interact with the neighboring less acidic but less hampered hydrogen atoms. More interestingly, the broadening of the ^1^H NMR signals in **3**, which are relative to the hydrogen atoms involved in hydrogen bonding interactions with the crown ether, suggests a continuous motion of the macrocycle along the H_10–13_ template site of the axle. In this *co*-conformational state, it seems that the DB24C8 tends to interact with the more acidic hydrogen atoms H_13_, while hindrance due to the rotations of the next amide and N-C_16_ bonds hampers this interaction. The ROESY ^1^H NMR spectrum of **3-Boc** presented in Figure 5a supports this hypothesis, this latter being illustrated in Figure 5b by the cartoon representations of the different conformers. It is particularly important to note the ROE correlation between the aromatic hydrogen atoms H_18–*cis*_ and the most acidic hydrogen atoms H_13–*cis*_. Highlighted by the cartoon representation of conformer **A** (Figure 5b), this close localization of the stopper with the template site should highly disfavor the expected hydrogen bonding interactions between the oxygen atoms of the DB24C8 and H_13_ due to steric repulsion. Noteworthy, no correlation exists between the same hydrogen atoms of the *trans* isomer (i.e., H_13–*trans*_ and H_18–*trans*_), agreeing the cartoon representations of the *trans* isomers **C** and **D**. The existence of conformer **A** is also demonstrated by the correlation between hydrogen atoms H_12–*cis*_ and H_16–*cis*_, while no correlation are noticed between H_12–*trans*_ and H_16–*trans*_ (see Supplementary Materials). The presence of conformers **A**-**B** are also confirmed by the absence of any correlation between the methylene hydrogen atoms H_15–*cis*_ and H_C–E_ belonging to the DB24C8 (see Supplementary Materials). This observation supports the fact that the *tert*-butylbenzyl stopper might prevent the DB24C8 from interacting with the close and more acidic hydrogen atoms H_13_ in the *cis* isomers **A** and **B**. It is completely the opposite for H_15–*trans*_, which strongly correlate with all the hydrogen atoms H_A–E_ of the DB24C8 (see Supplementary Materials). Finally, correlations between H_10–12_ with the methylene hydrogen atoms of the DB24C8 H_C–E_ (see Supplementary Materials) are consistent with the hydrogen-bonding interactions already stated with the help of the 1D ^1^H NMR spectra comparisons.

## 3. Conclusions

In conclusion, we have reported four new DB24C8-based molecular shuttles that contain either a secondary or a tertiary amide station. In the secondary amide series, deprotonation or deprotonation-carbamoylation of the best ammonium station led to a stable *co*-conformational state in which the DB24C8 interacts through hydrogen bonding with both the hydrogen atom linked to the nitrogen of the amide and the benzylic methylene hydrogens. The tertiary amide moiety proved to be a weaker station than the secondary one, although it could act as a secondary station in the case of a deprotonation-carbamoylation of the ammonium. In the case of a sole deprotonation, no shuttling of the DB24C8 around the tertiary amide motif occurred: the DB24C8 stayed around the amine moiety where it interacts through hydrogen bonds, albeit in a different manner than when it resides around the ammonium station. Actually, the DB24C8 prefers to interact with the benzylic methylene hydrogen atoms neighboring the ammonium rather than with the alkyl methylene group next to the ammonium, while it is the contrary after revealing the amine station. Eventually, deprotonation-carbamoylation resulted in a different behavior of the molecular shuttle in the tertiary amide series. In this case, the concealing of both the ammonium and amine station triggers the shuttling of the DB24C8 towards the amide site, although in a distinct manner than in the secondary amide series. Indeed, the tertiary amide moiety allows for a *cis*-*trans* isomerism at room temperature that hampers the axle at this site, preventing the DB24C8 from interacting through hydrogen bonds with the hydrogen atoms implied in the secondary amide series. In this case, the DB24C8 sits over the hydrogen atoms borne by the carbons in positions α, β, γ and δ of the carboxamide, where it interacts through hydrogen bonding interactions. More interestingly, due to steric hindrance generated by bonds rotations, the DB24C8 is not localized solely around the expected more acidic hydrogen atoms. It rather seems to adopt a continuous motion over 4 methylene groups. In summary, the DB24C8 can interact through hydrogen bonds with the following stations, albeit with the respective decreasing of affinity: (1) ammonium, (2) *N*-benzylic secondary amide, (3) amine, and (4) *N*-benzylic tertiary amide. New efficient molecular stations of very weak affinity for the macrocycle might be of interest for the conception of multi-stable molecular shuttles with fast shuttling.

## Supplementary Materials

Supplementary Materials are available online.
