# Supplementary material for: How Secondary and Tertiary Amide Moieties are Molecular Stations for Dibenzo-24-crown-8 in [2]Rotaxane Molecular Shuttles?
Source: Molecules. 2017 Nov 21;22(11):2017. doi: 10.3390/molecules22112017 (PMC6150268; doi:10.3390/molecules22112017)
Supplement: Supplementary file 1 [file molecules-22-02017-s001.pdf]

How secondary and tertiary amide moieties are molecular stations for dibenzo-24-crown-8 in [2]rotaxane molecular shuttles ?

Benjamin Riss-Yaw[1], Justine Morin[1], Caroline Clavel[1] and Frédéric Coutrot[1,*]

Supramolecular **M**achines and **AR**chitectures **T**eam, Institut des Biomolécules Max Mousseron (IBMM) UMR 5247 CNRS, Université Montpellier, ENSCM, case courrier 1706, Bâtiment Chimie (17), 3ème étage, Faculté des Sciences, Place Eugène Bataillon 34095 Montpellier cedex 5, France.

Tél : (+) 33 4 67 14 38 43 - Fax: (+) 33 4 67 63 10 46

E-mail: frederic.coutrot@umontpellier.fr, homepage: www.glycorotaxane.fr

Table of contents

Table of contents 1

1. Synthesis of [2]rotaxanes **2-H⁺**, **2** and **2-Boc**. 2

  1.1. General synthetic pathway 2

  1.2. Synthesis of [2]rotaxane **2-H⁺** 2

  1.3. Synthesis of [2]rotaxane **2** 3

  1.4. Synthesis of [2]rotaxane **2-Boc** 4

2. Synthesis of [2]rotaxanes **3-H⁺**, **3** and **3-Boc**. 4

  2.1. General synthetic pathway 4

  2.2. Synthesis of [2]rotaxane **3-H⁺** 5

  2.3. Synthesis of [2]rotaxane **3** 5

  2.4. Synthesis of [2]rotaxane **3-Boc** 6

  Selected 2D-ROESY ^{1}H spectrum portions (500MHz, CD_3CN, 298K) of rotaxane **3-Boc** 7

3. Synthesis of the uncomplexed threads **2u**, **2u-H⁺** and **2u-Boc** 8

  3.1. General synthetic pathway 8

  3.2. Synthesis of carbamate thread **2u-Boc** 8

  3.3. Synthesis of compound **2u-H⁺** 9

  3.4. Synthesis of compound **2u** 9

4. Synthesis of the uncomplexed threads **3u**, **3u-H⁺** and **3u-Boc** 10

  4.1. General synthetic pathway 10

  4.2. Synthesis of compound **3u-Boc** 10

  4.3. Synthesis of compound **3u-H⁺** 11

  4.4. Synthesis of compound **3u** 11

SPECTRA........ 12

General methods

All reactions were carried out under an atmosphere of argon unless otherwise indicated. All reagents were used as received without further purification. Dichloromethane was distilled over P_2O_5. Analytical thin-layer chromatography (TLC) was performed on silica gel 60 F254 plates. Compounds were visualized by dipping the plates in an ethanolic solution of 10% sulphuric acid, ninhydrine or an aqueous solution of $KMnO_4$, followed by heating. ^{1}H NMR and ^{13}C NMR spectra were obtained on a Bruker 500 Avance III spectrometer (respectively at 500.17 and 125.77 MHz). Chemical shifts of ^{1}H NMR and ^{13}C NMR are given by using CH_3CN as references (1.94 ppm respectively for ^{1}H specta, and 18.26 ppm respectively for ^{13}C spectra). ^{1}H assignments were deduced from 2D ^{1}H-^{1}H NMR COSY experiments and from 2D ^{1}H-^{1}H NMR ROESY experiments. ^{13}C assignments were deduced from 2D ^{13}C-^{1}H NMR HSQC experiments. Coupling constants (J) are reported in hertz (Hz). Standard abbreviations indicating multiplicity were used as follows: s (singlet), br (broad), d (doublet), t (triplet), q (quartet), quint (quintuplet), m (multiplet). Mass spectra (MS) and high-resolution mass spectra (HRMS) were recorded respectively on a ZQ Micromass apparatus and a Q-TOF Micro apparatus.

1. Synthesis of [2]rotaxanes 2-H⁺, 2 and 2-Boc.

1.1. General synthetic pathway

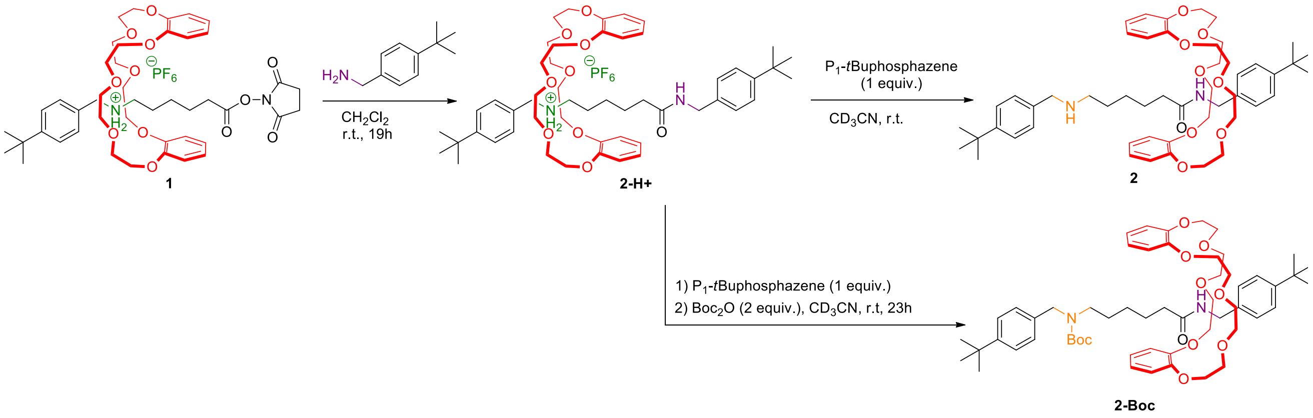

1.2. Synthesis of [2]rotaxane 2-H⁺

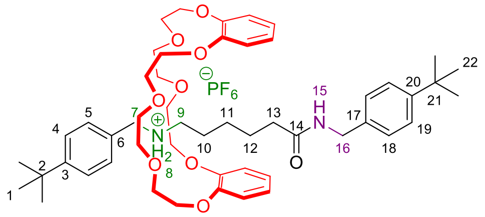

2-H⁺

To a solution of rotaxane building block **1** [1] (200 mg, 0.21 mmol, 1 equiv.) in dry CH_2Cl_2 (6 mL) was added 4-*tert*-butylbenzyl amine (103 mg, 0.62 mmol, 3.1 equiv.). The mixture was stirred for 19h at

[1] Synthesis and characterization of **1** have already been described by F. Coutrot *et al.* in *Chem. Eur. J.*, **2016**, 22, 8835-8847.

room temperature and evaporated to dryness. The crude was purified by chromatography on a lipophilic sephadex LH20 (CH_2Cl_2) to afford the pure rotaxane **2-H⁺** (143 mg, 53 %) as a white solid.

R_f: 0.65 (CH_2Cl_2/MeOH 9/1).
^1H NMR (500 MHz, CD_3CN, 298K): δ ppm = 7.37 (d, 2H, $^3J_{H19-H18}$ = 8.3 Hz, H_{19}), 7.27 (d, 2H, $^3J_{H5-H4}$ = 8.3 Hz, H_5), 7.21 (d, 2H, $^3J_{H4-H5}$ = 8.3 Hz, H_4), 7.18 (2d, 2H, $^3J_{H18-H19}$ = 8.3 Hz, H_{18}), 7.12 (br s, 2H, H_8), 6.91-6.86 (m, 8H, H_A H_B), 6.59 (br t, 2H, H_{15}), 4.55-4.50 (m, 2H, H_7), 4.25 (d, 2H, $^3J_{H16-H15}$ = 6.0 Hz, H_{16}), 4.18-4.12 & 4.08-4.03 (2m, 2X4H, H_C $H_{C'}$), 3.84-3.75 (m, 8H, H_D $H_{D'}$), 3.67-3.61 & 3.55-3.49 (2m, 2X4H, H_E $H_{E'}$), 3.33-3.26 (m, 2H, H_9), 1.92 (t, 2H, $^3J_{H13-H12}$ = 7.5 Hz, H_{13}), 1.52-1.43 (m, 2H, H_{10}), 1.38-1.31 (m, 2H, H_{12}), 1.29 (s, 9H, H_{22}), 1.23 (s, 9H, H_1), 1.15-1.06 (m, 2H, H_{11}).
^{13}C NMR (126 MHz, CD_3CN, 298K): δ ppm = 173.0 (C_{14}), 153.0 & 150.8 (C_3 C_{20}), 148.5 ($C_{IV\ arom\ DB24C8}$), 137.6 (C_{17}), 130.5 (C_5), 130.4 (C_6), 128.1 (C_{18}), 126.3 & 126.3 (C_4 C_{19}), 122.3 & 113.6 (C_A C_B), 71.6 (C_E $C_{E'}$), 71.1 (C_D $C_{D'}$), 69.0 (C_C $C_{C'}$), 52.8 (C_7), 49.5 (C_9), 43.1 (C_{16}), 36.2 (C_{13}), 35.1 & 35.0 (C_2 C_{21}), 31.5 (C_{22}), 31.4 (C_1), 27.0 (C_{10}), 26.6 (C_{11}), 25.6 (C_{12}).
HRMS (ESI): $[M-PF_6]^+$ calcd for $C_{52}H_{75}N_2O_9^+$: 871.5473, found: 871.5475.

1.3. Synthesis of [2]rotaxane 2

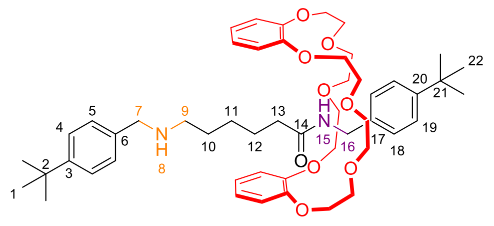

2

To a solution of rotaxane **2-H⁺** (18 mg, 0.017 mmol, 1 equiv.) in CD_3CN (0.6 mL) was added P1-*t*Buphosphazene (4.2 mg, 0.018 mmol, 1 equiv.), directly in the NMR tube. The rotaxane **2** (quantitative conversion) was characterized without being isolated.

^1H NMR (500 MHz, CD_3CN, 298K): δ ppm = 7.57 (d, 2H, $^3J_{H18-H19}$ = 8.3Hz, H_{18}), 7.34 (d, 2H, $^3J_{H4-H5}$ = 8.3Hz, H_4), 7.23 (br s, 1H, H_{15}), 7.21 (d, 2H, $^3J_{H19-H18}$ = 8.3Hz, H_5), 7.12 (d, 2H, $^3J_{H19-H18}$ = 8.3Hz, H_{19}), 6.89-6.83 (m, 8H, H_A H_B), 4.66 (br d, 2H, H_{16}), 4.12-4.07 & 4.03-3.98 (2m, 2x4H, H_C $H_{C'}$), 3.72-3.67 & 3.67-3.61 (2m, 2x4H, H_D $H_{D'}$), 3.62 (s, 2H, H_7), 3.48-3.42 & 3.23-3.17 (2m, 2x4H, H_E $H_{E'}$), 2.48-2.34 (m, 2H, H_9), 1.95 (br t, 2H, H_{13}), 1.43-1.35 (m, 2H, H_{12}), 1.30-1.22 (m, 2H, H_{10}), 1.29 (s, 9H, H_1), 1.25 & 1.24 (2s, 9H, H_{22}), 1.08-1.01 (m, 2H, H_{11}).
^{13}C NMR (126 MHz, CD_3CN, 298K): δ ppm = 173.1 (C_{14}), 150.3 & 149.4 & 149.3 (C_3 C_{20} $C_{IV\ arom\ DB24C8}$), 139.4 & 138.4 (C_6 C_{17}), 130.1 (C_{18}), 128.8 (C_5), 126.0 (C_4), 124.7 (C_{19}), 121.6 & 113.0 (C_A C_B), 71.1 (C_E $C_{E'}$), 70.3 (C_D $C_{D'}$), 69.1 (C_C $C_{C'}$), 54.0 (C_7), 50.0 (C_9), 43.8 (C_{16}), 36.8 (C_{13}), 35.0 & 34.8 (C_2 C_{21}), 32.5 (C_{22}), 31.7 & 31.6 (C_1), 30.6 (C_{10}), 27.9 (C_{11}), 26.0 (C_{12}).
HRMS (ESI): $[M+H]^+$ calcd for $C_{52}H_{75}N_2O_9^+$: 871.5473, found: 871.5477.

1.4. Synthesis of [2]rotaxane 2-Boc

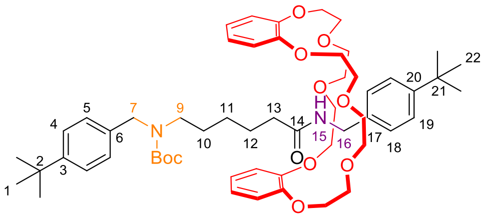

2-Boc

To a solution of rotaxane **2-H⁺** (67 mg, 0.066 mmol, 1 equiv.) in dry CH_2Cl_2 (1.2 mL) were successively added P1-*t*Buphosphazene (15.4 mg, 0.066 mmol, 1 equiv.) and Boc_2O (32 mg, 0.146 mmol, 2.2 equiv.). The mixture was stirred for 23h at room temperature before being evaporated to dryness. The crude was purified by chromatography on a lipophilic sephadex LH20 (CH_2Cl_2) to afford the pure rotaxane **2-Boc** (57 mg, 89 %) as a white solid.

R_f: 0.71 (CH_2Cl_2/MeOH 9/1).
^{1}H NMR (500 MHz, CD_3CN, 298K): δ ppm = 7.57 (d, 2H, , $^3J_{H18\text{-}H19}$ = 8.3Hz, H_{18}), 7.37 (d, 2H, $^3J_{H4\text{-}H5}$ = 8.3Hz, H_4), 7.24 (br t, 1H, H_{15}), 7.12 (d, 2H, , $^3J_{H5\text{-}H4}$ = 8.3Hz, H_5), 7.12 (d, 2H, $^3J_{H19\text{-}H18}$ = 8.3Hz, H_{19}), 6.89-6.83 (m, 8H, H_A H_B), 4.65 (br d, 2H, H_{16}), 4.28 (s, 2H, H_7), 4.12-4.07 & 4.03-3.98 (2m, 2x4H, H_C $H_{C'}$), 3.72-3.61 (m, 8H, H_D $H_{D'}$), 3.48-3.42 & 3.23-3.17 (2m, 2x4H, H_E $H_{E'}$), 3.01-2.93 (m, 2H, H_9), 1.97 (br t, 2H, H_{13}), 1.45-1.35 (m, 2H, H_{12}), 1.41 (br s, 9H, H_{Boc}), 1.35-1.26 (m, 2H, H_{10}), 1.30 (s, 9H, H_1), 1.24 (s, 9H, H_{22}), 1.02-0.94 (m, 2H, H_{11}).
^{13}C NMR (126 MHz, CD_3CN, 298K): δ ppm = 173.1 (C_{14}), 156.6 & 156.1 ($C_{IV\ CO\ Boc}$), 150.8 & 149.4 (C_3 C_{20}), 149.3 ($C_{IV\ arom\ DB24C8}$), 138.4 & 137.1 (C_{17} C_6), 130.1 (C_{18}), 128.0 (C_5), 126.2 (C_4), 124.7 (C_{19}), 121.6 & 113.0 (C_A C_B), 79.7 ($C_{IV\ Boc}$), 71.1 (C_E $C_{E'}$), 70.3 (C_D $C_{D'}$), 69.1 (C_C $C_{C'}$), 50.4 & 50.0 (C_7), 47.6 & 47.2 (C_9), 43.8 (C_{16}), 36.8 (C_{13}), 35.0 & 34.8 (C_{21} C_2), 31.7 & 31.6 (C_1 C_{22}), 29.0 & 28.6 & 28.4 (C_{Boc} C_{10}), 27.4 (C_{11}), 25.8 (C_{12}).
HRMS (ESI): $[M+H]^+$ calcd for $C_{57}H_{83}N_2O_{11}^+$: 971.5997, found: 971.5999.

2. Synthesis of [2]rotaxanes 3-H⁺, 3 and 3-Boc.

2.1. General synthetic pathway

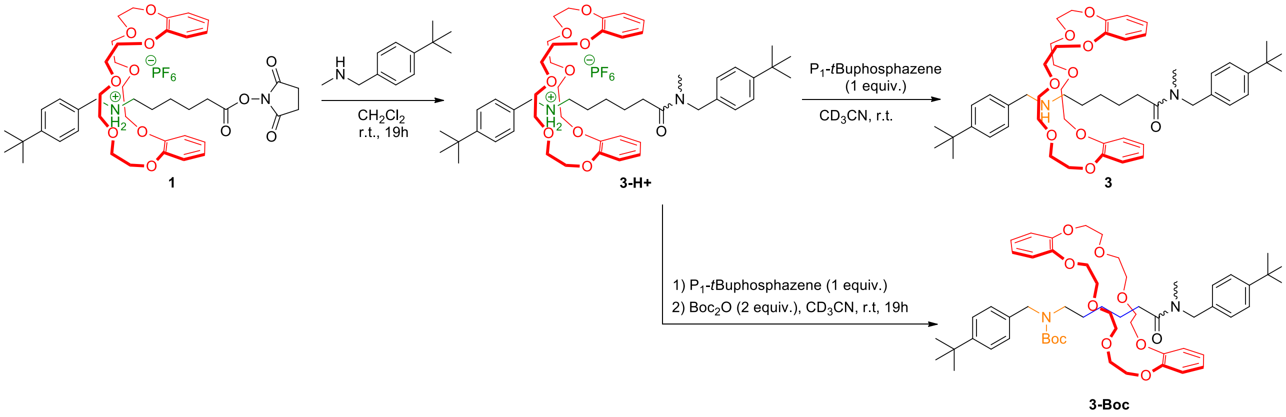

2.2. Synthesis of [2]rotaxane 3-H⁺

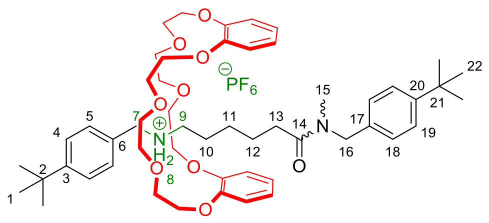

3-H⁺ (*trans*/*cis* 61/39)

To a solution of rotaxane building block **1**[2] (101 mg, 0.10 mmol, 1 equiv.) in dry CH_2Cl_2 (2 mL) was added the *N*-methyl-4-*tert*-butylbenzylamine (56 mg, 0.32 mmol, 3 equiv.). The mixture was stirred for 19h at room temperature and evaporated to dryness. The crude was purified by chromatography on a lipophilic sephadex LH20 (CH_2Cl_2) to afford the pure rotaxane **3-H⁺** (74 mg, 70 %) as a white solid.

R_f: 0.71 (CH_2Cl_2/MeOH 9/1).
^{1}H NMR (500 MHz, CD_3CN, 298K): δ ppm = 7.42 & 7.37 (2d, 2H, $^3J_{H19\text{-}H18}$ = 8.2 Hz, $H_{19\text{-cis}}$ $H_{19\text{-trans}}$), 7.28 (d, 2H, $^3J_{H5\text{-}H4}$ = 8.2 Hz, H_5), 7.22 (d, 2H, $^3J_{H4\text{-}H5}$ = 8.2 Hz, H_4), 7.17-7.07 (br s, 2H, H_8), 7.14 & 7.09 (2d, 2H, $^3J_{H18\text{-}H19}$ = 8.2 Hz, $H_{18\text{-trans}}$ $H_{18\text{-cis}}$), 6.92-6.84 (m, 8H, H_A H_B), 4.56-4.48 (m, 2H, H_7), 4.46 & 4.43 (2s, 2H, $H_{16\text{-}trans}$ $H_{16\text{-}cis}$), 4.19-4.11 & 4.09-4.01 (m, 8H, H_C $H_{C'}$), 3.83-3.76 (m, 8H, H_D $H_{D'}$), 3.69-3.60 & 3.57-3.48 (m, 8H, H_E $H_{E'}$), 3.38-3.31 & 3.31-3.24 (2m, 2H, H_9), 2.84 & 2.82 (2s, 3H, $H_{15\text{-}trans}$ $H_{15\text{-}cis}$), 2.08 & 2.06 (2br t, 2H, H_{13}), 1.57-1.47 & 1.47-1.41 (2m, 2H, $H_{10\text{-}trans}$ $H_{10\text{-}cis}$), 1.41-1.32 (m, 2H, H_{12}), 1.30 & 1.29 (2s, 9H, $H_{22\text{-}cis}$ $H_{22\text{-}trans}$), 1.23 (s, 9H, H_1), 1.15-1.07 & 1.06-0.99 (2m, 2H, $H_{11\text{-}trans}$ $H_{11\text{-}cis}$).
^{13}C NMR (126 MHz, CD_3CN, 298K): δ ppm = 173.3 & 173.1 (C_{14}), 153.0 & 151.2 & 150.9 (C_3 C_{20}), 148.5 ($C_{IV\text{ arom DB24C8}}$), 136.3 & 135.6 ($C_{17}$), 130.4 & 130.4 ($C_6$), 130.5 ($C_5$), 128.3 ($C_{18\text{-}trans}$), 127.1 ($C_{18\text{-}cis}$), 126.6 ($C_{19\text{-}cis}$), 126.3 ($C_4$ $C_{19\text{-}trans}$), 122.3 & 113.5 (C_A C_B), 71.6 & 71.6 (C_E $C_{E'}$), 71.1 & 71.1 (C_D $C_{D'}$), 69.0 (C_C $C_{C'}$), 53.3 (C_{16cis}), 52.8 & 52.8 (C_7), 50.4 ($C_{16\text{-}trans}$), 49.6 (C_9), 35.5 ($C_{15\text{-}trans}$), 35.1 & 35.1 & 35.0 (C_{21} C_2), 34.0 ($C_{15\text{-}cis}$), 33.4 & 33.1 ($C_{13\text{-}trans}$ $C_{13\text{-}cis}$), 31.6 & 31.4 (C_{22} C_1), 27.2 & 27.1 (C_{10}), 26.8 & 26.7 (C_{11}), 25.3 & 25.1 (C_{12}).
HRMS (ESI): $[M\text{-}PF_6]^+$ calcd for $C_{53}H_{77}N_2O_9^+$: 885.5629, found: 885.5632.

2.3. Synthesis of [2]rotaxane 3

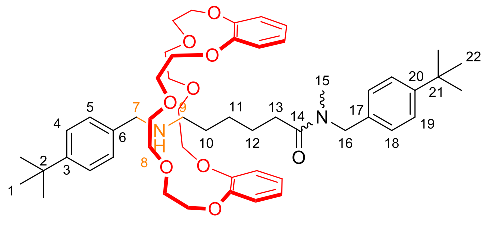

3 (*trans*/*cis* 59/41)

To a solution of rotaxane **3-H⁺** (56 mg, 0.054 mmol, 1 equiv.) in CD_3CN (0.6 mL) was added P1-*t*Buphosphazene (13 mg, 0.055 mmol, 1 equiv.), directly in the NMR tube. The rotaxane **3** (quantitative conversion) was characterized without being isolated.

[2] Synthesis and characterization of **1** have already been described by F. Coutrot *et al.* in *Chem. Eur. J.*, **2016**, 22, 8835-8847.

^{1}H NMR (500 MHz, CD_3CN, 298K): δ ppm = 7.36 & 7.34 (2br d, 2H, H_{19}), 7.21-7.15 (m, 4H, H_4 H_5), 7.10 & 7.00 (2br d, 2H, $H_{18\text{-}trans}$ $H_{18\text{-}cis}$), 6.89-6.82 (m, 8H, H_A H_B), 4.41 & 4.37 (2s, 2H, $H_{16\text{-}trans}$ $H_{16\text{-}cis}$), 4.14-4.02 (m, 8H, H_C $H_{C'}$), 3.81-3.77 & 3.77-3.73 (2m, 2x4H, H_D $H_{D'}$), 3.74 & 3.70 (2s, 2H, H_7), 3.62-3.56 & 3.56-3.47 (2m, 8H, H_E $H_{E'}$), 2.75 & 2.72 (2s, 3H, $H_{15\text{-}trans}$ $H_{15\text{-}cis}$), 2.71-2.61 (m, 2H, H_9), 2.22 (br t, 2H, H_{13}), 1.58-1.43 (m, 4H, H_{12} H_{10}), 1.34-1.26 (m, 2H, H_{11}), 1.29 & 1.28 (2s, 9H, H_{22}), 1.24 (s, 9H, H_1).
^{13}C NMR (126 MHz, CD_3CN, 298K): δ ppm = 174.6 & 174.4 (C_{14}), 150.9 & 150.7 & 149.8 (C_3 C_{20}), 149.2 ($C_{IV\ arom\ DB24C8}$), 136.5 & 135.9 (C_6 C_{17}), 129.3 (C_5), 128.3 ($C_{18\text{-}trans}$), 127.2 ($C_{18\text{-}cis}$), 126.5 & 126.3 (C_{19}), 125.6 (C_4), 121.7 & 113.3 & 113.2 (C_A C_B), 71.4 (C_E $C_{E'}$), 70.4 (C_D $C_{D'}$), 69.0 & 69.0 (C_C $C_{C'}$), 53.5 (C_7), 53.3 ($C_{16\text{-}cis}$), 50.7 ($C_{16\text{-}trans}$), 50.3 (C_9), 35.4 ($C_{15\text{-}trans}$), 35.0 & 35.0 & 34.9 (C_2 C_{21}), 34.1 & 33.9 ($C_{13\text{-}trans}$ $C_{13\text{-}cis}$), 33.7 ($C_{15\text{-}cis}$), 31.6 & 31.6 (C_{22} C_1), 29.6 (C_{10}), 27.7 & 27.6 (C_{11}), 26.1 & 25.7 (C_{12}).
HRMS (ESI): $[M+H]^+$ calcd for $C_{52}H_{75}N_2O_9^+$: 885.5629, found: 885.5632.

2.4. Synthesis of [2]rotaxane 3-Boc

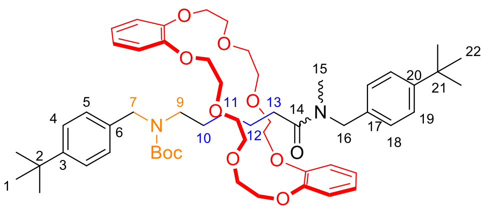

3-Boc (*trans/cis* 56/44)

To a solution of rotaxane **3-H$^+$** (56 mg, 0.054 mmol, 1 equiv.) in dry CH_2Cl_2 (1 mL) were successively added P1-*t*Buphosphazene (13 mg, 0.055 mmol, 1 equiv.) and Boc_2O (26 mg, 0.119 mmol, 2.2 equiv.). The mixture was stirred for 19h at room temperature before being evaporated to dryness. The crude was purified by chromatography on a lipophilic sephadex LH20 (CH_2Cl_2) to afford the pure rotaxane **3-Boc** (50 mg, 93 %) as a white solid.

R_f: 0.74 (CH_2Cl_2/MeOH 9/1).
^{1}H NMR (500 MHz, CD_3CN, 298K): δ ppm = 7.31-7.21 (m, 4H, H_4 H_{19}), 7.04 & 6.88 (2br d, 2H, $H_{18\text{-}trans}$ $H_{18\text{-}cis}$), 6.95 (br d, 2H, H_5), 6.89-6.79 (m, 8H, H_A H_B), 4.34 & 4.31 (2s, 2H, $H_{16\text{-}trans}$ $H_{16\text{-}cis}$), 4.10 (br s, 2H, H_7), 4.15-4.06 & 4.06-3.99 (2m, 8H, H_C $H_{C'}$), 3.83-3.77 & 3.77-3.65 (2m, 8H, H_D $H_{D'}$), 3.61-3.56 & 3.56-3.50 (2m, 8H, H_E $H_{E'}$), 3.08-2.91 (m, 2H, H_9), 2.66 & 2.61 (2s, 3H, $H_{15\text{-}trans}$ $H_{15\text{-}cis}$), 2.56-2.43 (m, 2H, H_{13}), 1.96-1.82 (m, 2H, H_{12}), 1.73-1.62 (m, 2H, H_{10}), 1.58-1.46 (m, 2H, H_{11}), 1.34 (br s, 9H, H_{Boc}), 1.29 & 1.28 & 1.27 & 1.27 (4s, 18H, H_1 H_{22}).
^{13}C NMR (126 MHz, CD_3CN, 298K): δ ppm = 176.3 & 176.0 (C_{14}), 156.8 & 156.2 ($C_{IV\ CO\ Boc}$), 150.6 & 150.5 & 150.3 & 149.8 (C_3 C_{20}), 149.4 & 149.3 ($C_{IV\ arom\ DB24C8}$), 137.4 & 137.2 & 136.6 & 136.1 (C_6 C_{17}), 128.2 ($C_{18\text{-}trans}$), 128.0 (C_5), 127.2 ($C_{18\text{-}cis}$), 126.3 & 126.2 ($C_{19\text{-}cis}$ $C_{19\text{-}trans}$), 126.0 (C_4), 121.4 & 121.4 & 113.0 & 113.0 (C_A C_B), 79.2 ($C_{IV\ Boc}$), 71.7 & 71.6 (C_E $C_{E'}$), 70.6 & 70.5 (C_D $C_{D'}$), 69.2 & 69.1 (C_C $C_{C'}$), 53.1 ($C_{16\text{-}cis}$), 50.4 ($C_{16\text{-}trans}$), 49.4 & 48.9 (C_7), 48.1 & 47.3 (C_9), 35.3 ($C_{15\text{-}trans}$), 35.0 & 35.0 (C_2 C_{17}), 34.7 & 34.4 ($C_{13\text{-}trans}$ $C_{13\text{-}cis}$), 33.2 ($C_{15\text{-}cis}$), 31.6 & 31.6 (C_{22} C_1), 28.7 (C_{Boc}), 28.3 & 27.8 (C_{10}), 27.4 & 27.1 & 27.1 & 27.0 (C_{11}), 26.0 & 25.8 & & 25.6 & 25.4 (C_{12}).
HRMS (ESI): $[M+H]^+$ calcd for $C_{58}H_{85}N_2O_{11}^+$: 985.6153, found: 985.6163.

Selected 2D-ROESY ^{1}H spectrum portions (500MHz, CD_3CN, 298K) of rotaxane **3-Boc**

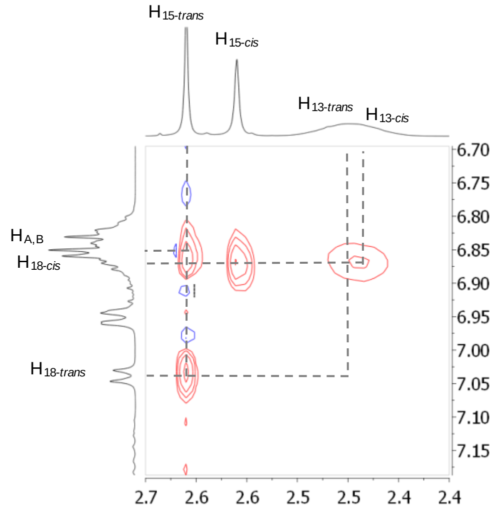

ROE correlation between the aromatic hydrogen atoms $H_{18\text{-}cis}$ and the most acidic hydrogen atoms $H_{13\text{-}cis}$. The same correlation is not observed for the *trans* isomers.

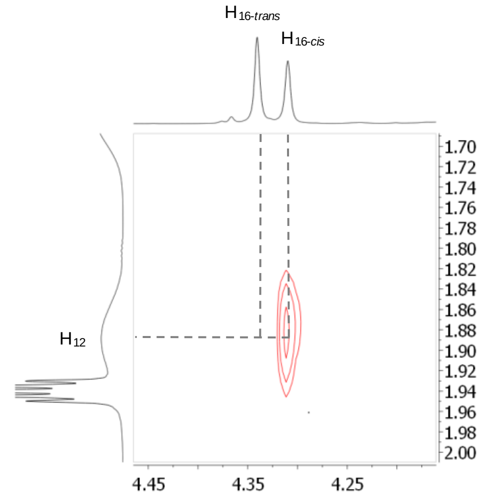

ROE correlation between hydrogen atoms $H_{16\text{-}cis}$ and hydrogen atoms $H_{12\text{-}cis}$. The same correlation is not observed for the *trans* isomers.

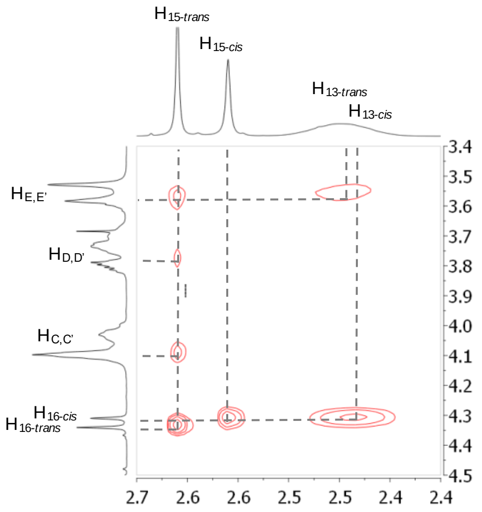

ROE correlation between the hydrogen atoms $H_{15\text{-}trans}$ and the hydrogen atoms $H_{C\text{-}E}$ belonging to the DB24C8. The same correlations are not observed for the *cis* isomers.

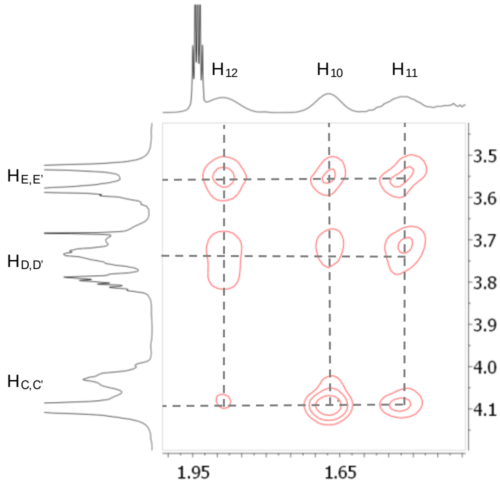

ROE correlation between the hydrogen atoms $H_{10\text{-}12}$ and the hydrogen atoms $H_{C\text{-}E}$ belonging to the DB24C8.

3. Synthesis of the uncomplexed threads 2u, 2u-H⁺ and 2u-Boc

3.1. General synthetic pathway

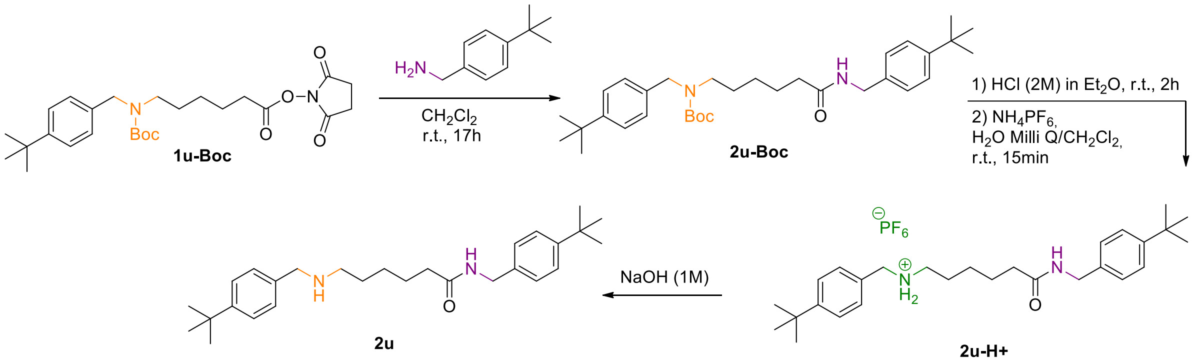

3.2. Synthesis of carbamate thread 2u-Boc

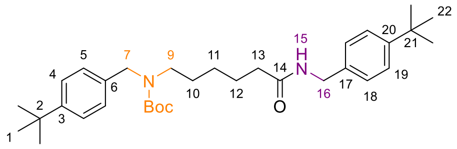

2u-Boc

To a solution of carbamate building block **1u-Boc**[3] (320 mg, 0.67 mmol, 1 equiv.) in dry CH_2Cl_2 (20 mL) was added the 4-*tert*-butylbenzylamine (220 mg, 1.37 mmol, 2 equiv.). The mixture was stirred for 17h at room temperature before being washed with an aqueous 1M solution of HCl (2x25 mL). The combined aqueous layers were extracted with CH_2Cl_2 (3x25 mL). Finally, the combined organic layers were washed with an aqueous 1M solution of NaOH (3x25 mL), dried over $MgSO_4$, filtrated and evaporated to afford the pure carbamate thread **2u-Boc** (347 mg, 98 %) as a colorless oil.

R_f: 0.82 (CH_2Cl_2/MeOH 9/1).
^{1}H NMR (500 MHz, CD_3CN, 298K): δ ppm = 7.37 & 7.35 (2br d, 4H, H_{19} H_4), 7.18 & 7.17 (2br d, 4H, H_{18} H_5), 6.77 (br s, 1H, H_{15}), 4.35 (s, 2H, H_7), 4.27 (d, 2H, $^3J_{H16\text{-}H15}$ = 6.1Hz, H_{16}), 3.13 (br t, 2H, H_9), 2.12 (t, 2H, $^3J_{H13\text{-}H12}$ = 7.4Hz, H_{13}), 1.59-1.52 (m, 2H, H_{12}), 1.52-1.43 (m, 2H, H_{10}), 1.43 (br s, 9H, H_{Boc}), 1.29 & 1.28 (2s, 18H, H_{22} H_1), 1.29-1.20 (m, 2H, H_{11}).
^{13}C NMR (126 MHz, CD_3CN, 298K): δ ppm = 173.5 (C_{14}), 156.7 & 156.3 ($C_{IV\ CO\ Boc}$), 150.9 & 150.7 (C_{20} C_3), 137.7 & 137.1 (C_{17} C_6), 128.1 & 128.0 (C_5 C_{18}), 126.3 (C_4 C_{19}), 79.8 ($C_{IV\ Boc}$), 50.5 & 50.1 (C_7), 47.3 & 47.1 (C_9), 43.0 (C_{16}), 36.7 (C_{13}), 35.0 (C_{21} C_2), 31.5 (C_1 C_{22}), 28.6 (C_{Boc}), 28.3 (C_{10}), 27.0 (C_{11}), 26.2 (C_{12}).
HRMS (ESI): $[M+H]^+$ calcd for $C_{33}H_{51}N_2O_3^+$: 523.3900, found: 523.3903.

---

[3] Synthesis and characterization of **1u-Boc** have already been described by F. Coutrot *et al.* in *Chem. Eur. J.*, **2016**, 22, 8835-8847.

3.3. Synthesis of compound 2u-H+

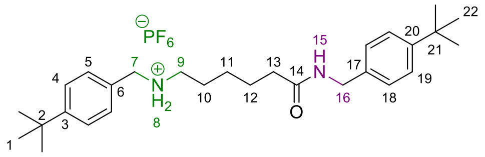

2u-H+

A solution of thread **2u-Boc** (284 mg, 0.56 mmol, 1 equiv.) in 2M HCl/Et_2O (5 mL) was stirred for 2h at 20°C until TLC analysis revealed no trace of starting material. The mixture was evaporated to dryness and the residue was partitioned between CH_2Cl_2 (3 mL) and Milli Q H_2O (1 mL). NH_4PF_6 (275 mg, 1.66 mmol, 3 equiv.) was added and the mixture was vigorously stirred for 15 minutes. The aqueous layer was extracted with CH_2Cl_2 (3x10 mL) and the combined organic layers were dried over $MgSO_4$ and concentrated to afford compound **2u-H+** (282 mg, 89 %) as a yellow solid without further purification.

R_f: 0.58 (CH_2Cl_2/MeOH 9/1).
^{1}H NMR (500 MHz, CD_3CN, 298K): δ ppm = 7.47 (br d, 2H, $^3J_{H4-H5}$ = 8.4 Hz, H_4), 7.39 (br d, 2H, $^3J_{H5-H4}$ = 8.4 Hz, H_5), 7.37 (br d, 2H, $^3J_{H19-H18}$ = 8.4 Hz, H_{19}), 7.28 (br s, 1H, H_8), 7.17 (br d, 2H, $^3J_{H18-H19}$ = 8.4 Hz, H_{18}), 6.95 (br s, 1H, H_{15}), 4.27 (d, 2H, $^3J_{H16-H15}$ = 6.1 Hz, H_{16}), 4.13-4.06 (m, 2H, H_7), 3.10-3.00 (m, 2H, H_9), 2.20 (t, 2H, $^3J_{H13-H12}$ = 7.1 Hz, H_{13}), 1.73-1.64 (m, 2H, H_{10}), 1.62-1.53 (m, 2H, H_{12}), 1.41-1.33 (m, 2H, H_{11}), 1.30 & 1.29 (2s, 18H, H_{22} H_1).
^{13}C NMR (126 MHz, CD_3CN, 298K): δ ppm = 174.1 (C_{14}), 153.7 & 150.9 (C_3 C_{20}), 137.7 (C_{17}), 130.8 (C_5), 128.8 (C_6), 128.0 (C_{18}), 126.9 (C_4), 126.3 (C_{19}), 52.1 (C_7), 48.2 (C_9), 43.2 (C_{16}), 35.6 (C_{13}), 35.3 & 35.0 (C_{21} C_2), 31.5 & 31.3 (C_{22} C_1), 25.9 (C_{11}), 25.7 (C_{10}), 24.4 (C_{12}).
HRMS (ESI): $[M-PF_6]^+$ calcd for $C_{28}H_{43}N_2O^+$: 423.3375, found: 423.3367.

3.4. Synthesis of compound 2u

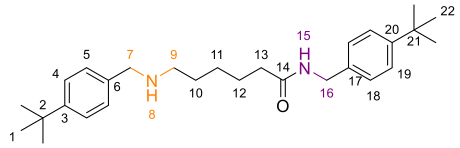

2u

A solution of compound **2u-H+** (182 mg, 0.32 mmol) in CH_2Cl_2 (5 mL) was washed with an aqueous 1M solution of NaOH (5 mL). The aqueous layer was extracted with CH_2Cl_2 (3x5 mL) and the combined organic layers were dried over $MgSO_4$ and evaporated to afford compound **2u** (137 mg, quantitative yield) as an orange oil.

R_f: 0.39 (CH_2Cl_2/MeOH 9/1).
^{1}H NMR (500 MHz, CD_3CN, 298K): δ ppm = 7.36 (d, 4H, $^3J_{H19-H18}$ = 8.1Hz $^3J_{H4-H5}$ = 8.1Hz, H_{19} H_4), 7.24 (d, 2H, $^3J_{H5-H4}$ = 8.1Hz, H_5), 7.18 (d, 2H, $^3J_{H18-H19}$ = 8.1Hz, H_{18}), 6.77 (br s, 1H, H_{15}), 4.27 (d, 2H, $^3J_{H9-H10}$ = 6.0Hz, H_{16}), 3.70 (s, 2H, H_7), 2.55 (t, 2H, $^3J_{H9-H10}$ = 7.0Hz, H_9), 2.15 (t, 2H, $^3J_{H13-H12}$ = 7.4Hz, H_{13}), 1.61-1.53 (m, 2H, H_{12}), 1.51-1.43 (m, 2H, H_{10}), 1.36-1.28 (m, 2H, H_{11}), 1.29 & 1.29 (2s, 18H, H_{22}, H_1).
^{13}C NMR (126 MHz, CD_3CN, 298K): δ ppm = 173.6 (C_{14}), 150.7 & 150.5 (C_3 C_{20}), 138.7 & 137.7 (C_{17} C_6), 128.8 (C_5), 128.0 (C_{18}), 126.2 & 126.1 (C_4 C_{19}), 53.9 (C_7), 49.8 (C_9), 43.0 (C_{16}), 36.7 (C_{13}), 35.0 (C_2 C_{21}), 31.6 & 31.5 (C_1 C_{22}), 30.2 (C_{10}), 27.5 (C_{11}), 26.3 (C_{12}).
HRMS (ESI): $[M+H]^+$ calcd for $C_{28}H_{43}N_2O^+$: 423.3375, found: 423.3375.

4. Synthesis of the uncomplexed threads 3u, 3u-H+ and 3u-Boc

4.1. General synthetic pathway

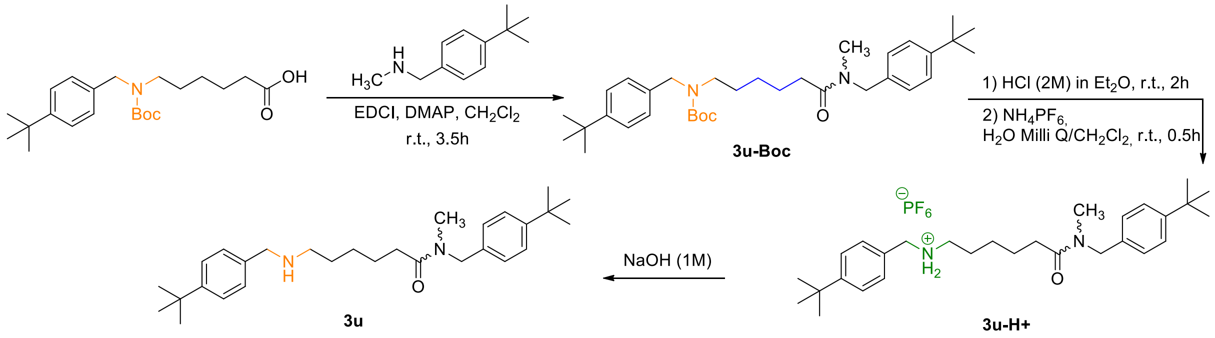

4.2. Synthesis of compound 3u-Boc

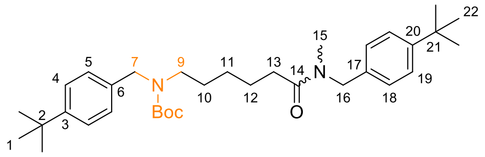

3u-Boc (*trans/cis* 62/38)

To a 0.02 M solution of carboxylic acid starting material[4] (320 mg, 0.85 mmol, 1 equiv.) and *N*-methyl-4-*tert*-butylbenzylamine (150 mg, 0.85 mmol, 1 equiv.) in dry amylene-stabilized CH_2Cl_2 were added EDCI (243 mg, 1.27 mmol, 1.5 equiv.) and DMAP (362 mg, 2.96 mmol, 3.5 equiv.). The mixture was stirred for 3h30 at room temperature before being washed with an aqueous 1M solution of HCl (15 mL). The aqueous layer was extracted with CH_2Cl_2 (15 mL) and the combined organic layers were washed with an aqueous 1M solution of NaOH (3x15 mL), dried over $MgSO_4$ and concentrated. The residue was purified by silica gel chromatography (PE/EtOAc 1/1) to give thread **3u-Boc** (250 mg, 55%) as orange oil.

R_f: 0.89 (CH_2Cl_2/MeOH 9/1).
^{1}H NMR (500 MHz, CD_3CN, 298K): δ ppm = 7.42-7.34 (m, 4H, H_{19} H_4), 7.19-7.09 (m, 4H, H_5 H_{18}), 4.50 & 4.48 (2s, 2H, H_{16}), 4.37 & 4.34 (2s, 2H, H_7), 3.19-3.08 (m, 2H, H_9), 2.90 & 2.83 (2s, 3H, $H_{15\text{-}trans}$ $H_{15\text{-}cis}$), 2.33 & 2.31 (2br t, 2H, $H_{13\text{-}trans}$ $H_{13\text{-}cis}$), 1.62-1.54 (m, 2H, H_{12}), 1.54-1.48 (m, 2H, H_{10}), 1.48-1.37 (m, 9H, H_{Boc}), 1.29 (s, 18H, H_{22} H_1), 1.33-1.19 (m, 2H, H_{11}).
^{13}C NMR (126 MHz, CD_3CN, 298K): δ ppm = 173.7 & 173.5 (C_{14}), 156.7 & 156.3 ($C_{IV\ CO\ Boc}$), 151.2 & 150.9 (C_3 C_{20}), 137.1 & 136.3 & 135.7 (C_{17} C_6), 128.2 & 128.1 & 127.2 (C_5 C_{18}), 126.6 & 126.3 & 126.2 (C_4 C_{19}), 79.8 ($C_{IV\ Boc}$), 53.4 ($C_{16\text{-}cis}$), 50.8 ($C_{16\text{-}trans}$), 50.5 & 50.1 (C_7), 47.4 & 47.1 (C_9), 35.5 ($C_{15\text{-}trans}$), 35.0 (C_{21} C_2), 34.0 ($C_{15\text{-}cis}$), 33.7 & 33.4 ($C_{13\text{-}trans}$ $C_{13\text{-}cis}$), 31.6 (C_{22} C_1), 28.8 & 28.4 (C_{10}), 28.6 (C_{Boc}), 27.2 (C_{11}), 25.8 & 25.6 (C_{12}).
HRMS (ESI): $[M+H]^+$ calcd for $C_{34}H_{53}N_2O_3^+$: 537.4056, found: 537.4058.

[4] Synthesis and characterization of carboxylic acid starting material have already been described by F. Coutrot *et al.* in *Chem. Eur. J.*, **2016**, 22, 8835-8847.

4.3. Synthesis of compound 3u-H+

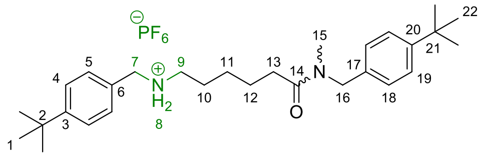

3u-H+ (*trans/cis* 64/36)

A solution of compound **3u-Boc** (206 mg, 0.384 mmol, 1 equiv.) in 2M HCl/Et_2O (10 mL) was stirred for 2h at 20°C until TLC analysis revealed no trace of starting material. The mixture was evaporated to dryness and the residue was partitioned between CH_2Cl_2 (4 mL) and MilliQ H_2O (2 mL). NH_4PF_6 (42 mg, 1.150 mmol, 3 equiv.) was added and the mixture was vigorously stirred for 30 minutes. The aqueous layer was extracted with CH_2Cl_2 (3x10 mL) and the combined organic layers were dried over $MgSO_4$ and concentrated to afford compound **3u-H+** (165 mg, 74%) without further purification.

R_f: 0.71 (CH_2Cl_2/MeOH 9/1).
^{1}H NMR (500 MHz, CD_3CN, 298K): δ ppm = 7.47-7.36 (m, 6H, H_4 H_5 H_{19}), 7.12 (d, 2H, $^3J_{H18-H19}$ = 8.1 Hz, H_{18}), 4.50 & 4.46 (2s, 2H, H_{16-cis} $H_{16-trans}$), 4.09 (s, 2H, H_7), 3.07 & 3.02 (2t, 2H, $^3J_{H9-H10}$ = 7.2 Hz, $H_{9-trans}$ H_{9-cis}), 2.91 & 2.81 (2s, 3H, $H_{15-trans}$ H_{15-cis}), 2.38 & 2.35 (2t, 2H, $^3J_{H13-H12}$ = 6.5 Hz, $H_{13-trans}$ H_{13-cis}), 1.79-1.69 (m, 2H, H_{10}), 1.58-1.49 (m, 2H, H_{12}), 1.42-1.30 (m, 2H, H_{11}), 1.29 & 1.25 (2s, 18H, H_1 H_{22}).
^{13}C NMR (126 MHz, CD_3CN, 298K): δ ppm = 174.4 & 174.3 (C_{14}), 153.5 & 153.5 & 151.4 & 151.1 (C_3 C_{20}), 135.6 & 135.1 (C_{17}), 130.9 (C_5), 129.1 (C_6), 128.3 & 127.3 & 126.8 & 126.7 & 126.7 & 126.4 (C_4 C_{18} C_{19}), 53.3 (C_{16-cis}), 52.0 & 51.9 (C_7), 51.2 ($C_{16-trans}$), 47.9 & 47.8 (C_9), 35.5 ($C_{15-trans}$), 35.2 & 35.2 & 35.1 & 35.0 (C_{21} C_2), 34.2 (C_{15-cis}), 32.9 & 32.8 ($C_{13-trans}$ C_{13-cis}), 31.5 & 31.4 (C_{22} C_1), 25.9 (C_{11-cis}), 25.7 ($C_{11-trans}$), 25.7 (C_{10-cis}), 25.4 ($C_{10-trans}$), 23.8 (C_{12-cis}), 23.1 ($C_{12-trans}$).
HRMS (ESI): $[M-PF_6]^+$ calcd for $C_{29}H_{45}N_2O^+$: 437.3532, found: 437.3529.

4.4. Synthesis of compound 3u

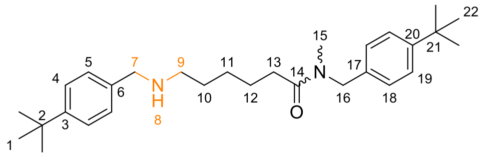

3u (*trans/cis* 62/38)

A solution of compound **3u-H+** (131 mg, 0.034 mmol) in CH_2Cl_2 (5 mL) was washed with an aqueous solution of NaOH 1M (5 mL). The aqueous layer was extracted with CH_2Cl_2 (3x5 mL) and the combined organic layers were dried over $MgSO_4$ and evaporated to afford compound **3u** (95 mg, quantitative yield) as an orange oil.

R_f: 0.57 (CH_2Cl_2/MeOH 9/1).
^{1}H NMR (500 MHz, CD_3CN, 298K): δ ppm = 7.41-7.33 (m, 4H, H_{19} H_4), 7.26-7.21 (m, 2H, H_5), 7.14 & 7.12 (2br d, 2H, H_{18}), 4.51 & 4.48 (2s, 2H, H_{16-cis} $H_{16-trans}$), 3.69 & 3.67 (2s, 2H, H_7), 2.90 & 2.83 (2s, 3H, $H_{15-trans}$ H_{15-cis}), 2.55 & 2.51 (2t, 2H, $^3J_{H9-H10}$ = 7.0Hz, $H_{9-trans}$ H_{9-cis}), 2.36 & 2.33 (2br t, 2H, $H_{13-trans}$ H_{13-cis}), 1.63-1.51 (m, 2H, H_{12}), 1.53-1.58 (m, 2H, H_{10}), 1.41-1.30 (m, 2H, H_{11}), 1.29 (s, 18H, H_1 H_{22}).
^{13}C NMR (126 MHz, CD_3CN, 298K): δ ppm = 173.8 & 173.6 (C_{14}), 151.2 & 150.8 & 150.4 (C_3 C_{20}), 139.1 & 136.3 & 135.7 (C_{17} C_6), 128.8 (C_5), 128.3 & 127.2 (C_{18}), 126.6 & 126.3 & 126.0 (C_4 C_{19}), 54.1 & 54.0 (C_7), 53.4 (C_{16-cis}), 50.9 ($C_{16-trans}$), 50.0 & 49.9 (C_9), 35.6 ($C_{15-trans}$), 35.1 & 35.0 (C_{21} C_2), 34.1 (C_{15-cis}), 33.9 & 33.5 ($C_{13-trans}$ C_{13-cis}), 31.8 & 31.7 (C_{22} C_1), 30.6 & 30.6 (C_{10}), 27.9 & 27.8 (C_{11}), 26.1 & 25.8 (C_{12}).
HRMS (ESI): $[M+H]^+$ calcd for $C_{29}H_{45}N_2O^+$: 437.3532, found: 437.3531.

SPECTRA

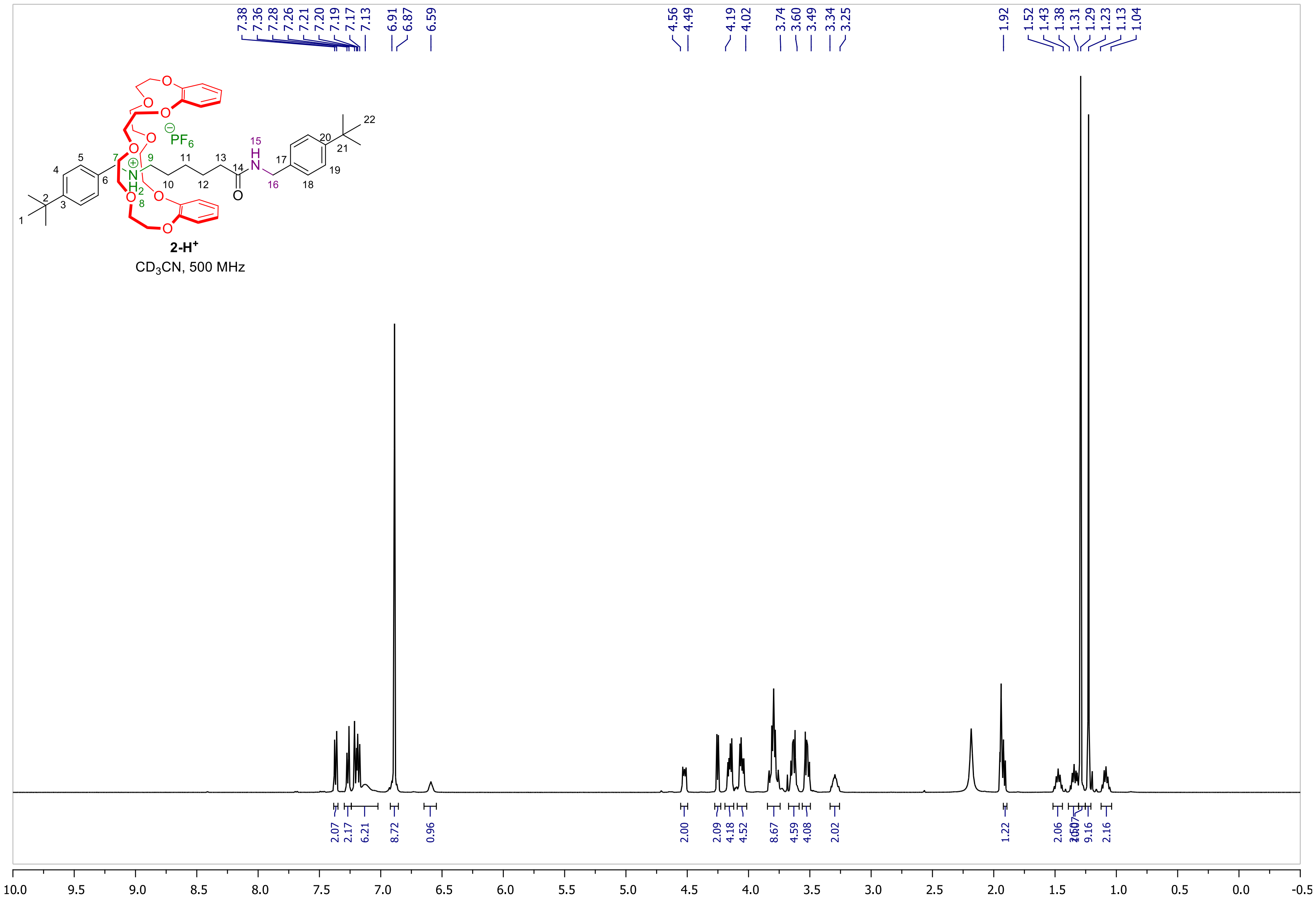

2-H+
CD3CN, 500 MHz
PF6
7.38
7.36
7.28
7.26
7.21
7.20
7.19
7.17
7.13
6.91
6.87
6.59
4.56
4.49
4.19
4.02
3.74
3.60
3.49
3.34
3.25
1.92
1.52
1.43
1.38
1.31
1.29
1.23
1.13
1.04
2.07
2.17
6.21
8.72
0.96
2.00
2.09
4.18
4.52
8.67
4.59
4.08
2.02
1.22
2.06
9.16
2.16
10.0
9.5
9.0
8.5
8.0
7.5
7.0
6.5
6.0
5.5
5.0
4.5
4.0
3.5
3.0
2.5
2.0
1.5
1.0
0.5
0.0
-0.5

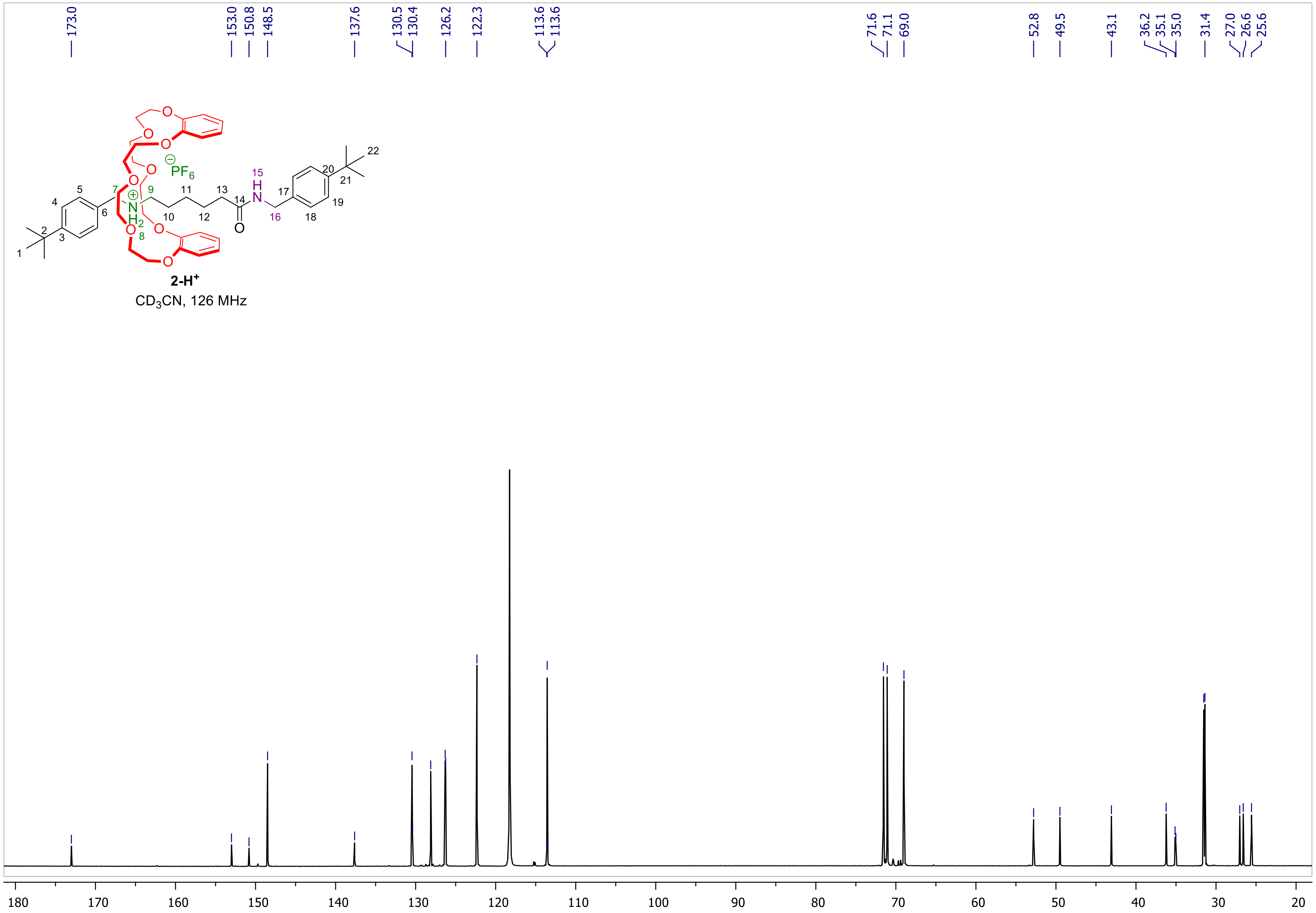

173.0
153.0
150.8
148.5
137.6
130.5
130.4
126.2
122.3
113.6
113.6
71.6
71.1
69.0
52.8
49.5
43.1
36.2
35.1
35.0
31.4
27.0
26.6
25.6
PF6
2-H+
CD3CN, 126 MHz
180
170
160
150
140
130
120
110
100
90
80
70
60
50
40
30
20

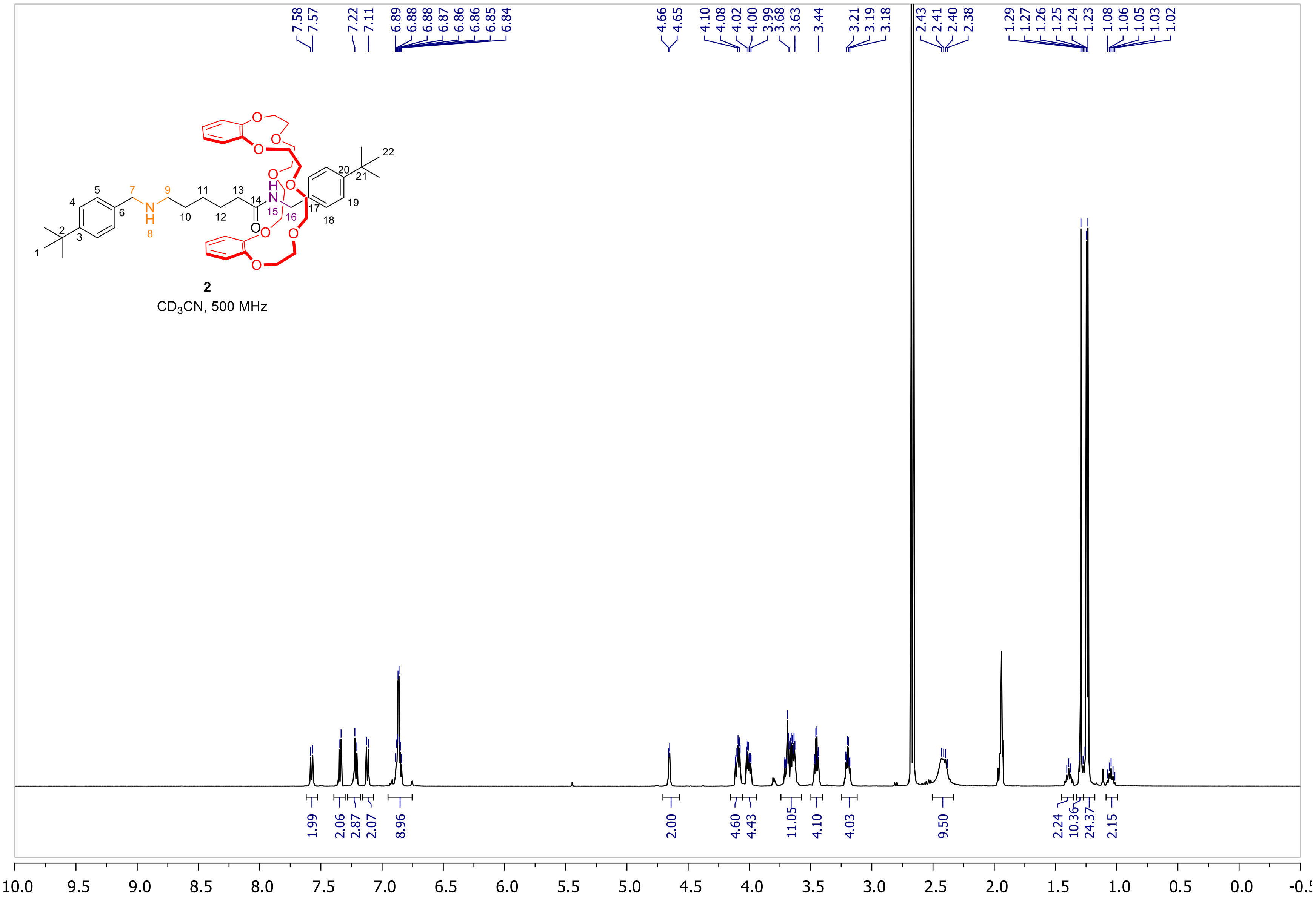
2
CD3CN, 500 MHz

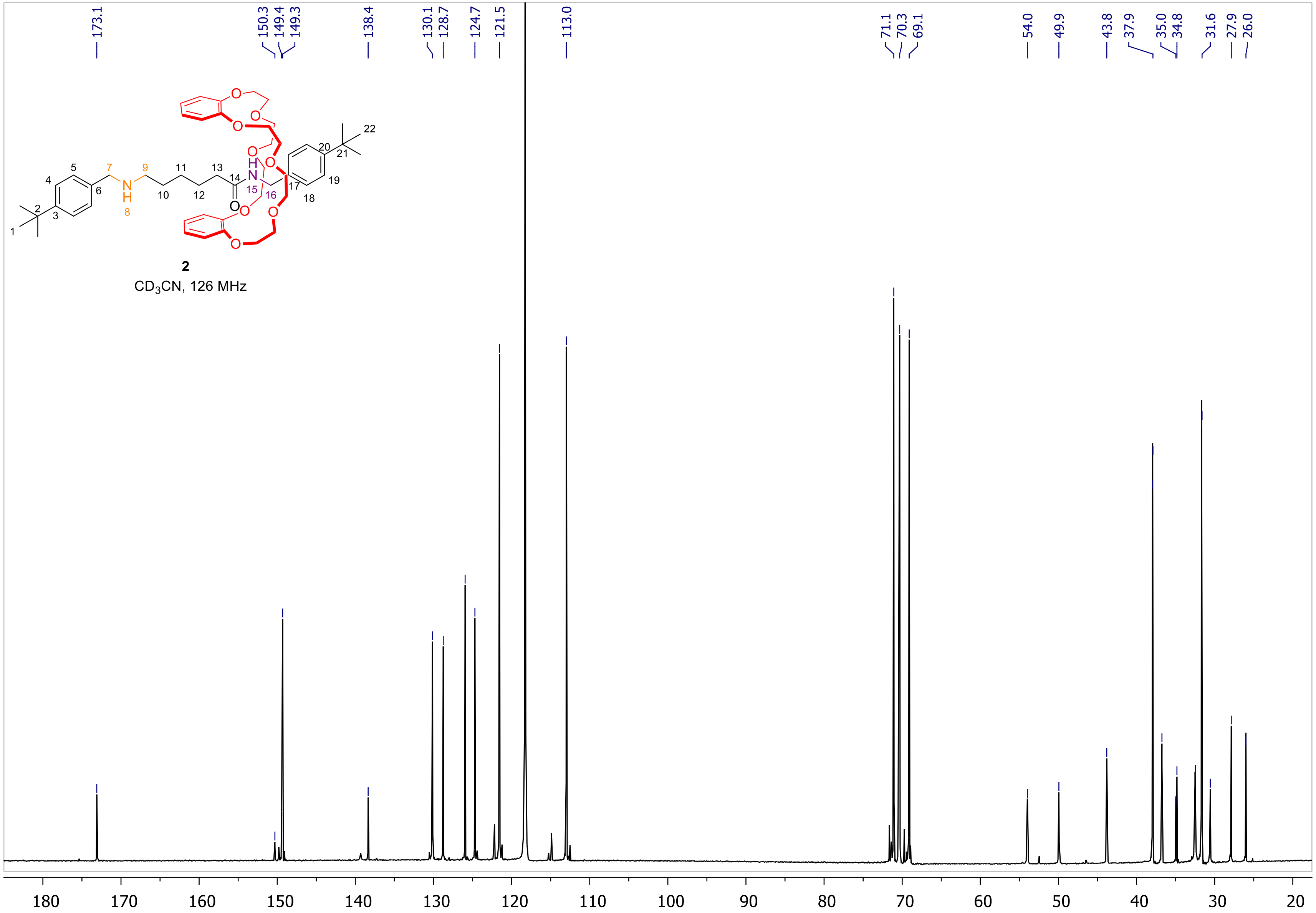
173.1
150.3
149.4
149.3
138.4
130.1
128.7
124.7
121.5
113.0
71.1
70.3
69.1
54.0
49.9
43.8
37.9
35.0
34.8
31.6
27.9
26.0
2
CD3CN, 126 MHz
180
170
160
150
140
130
120
110
100
90
80
70
60
50
40
30
20

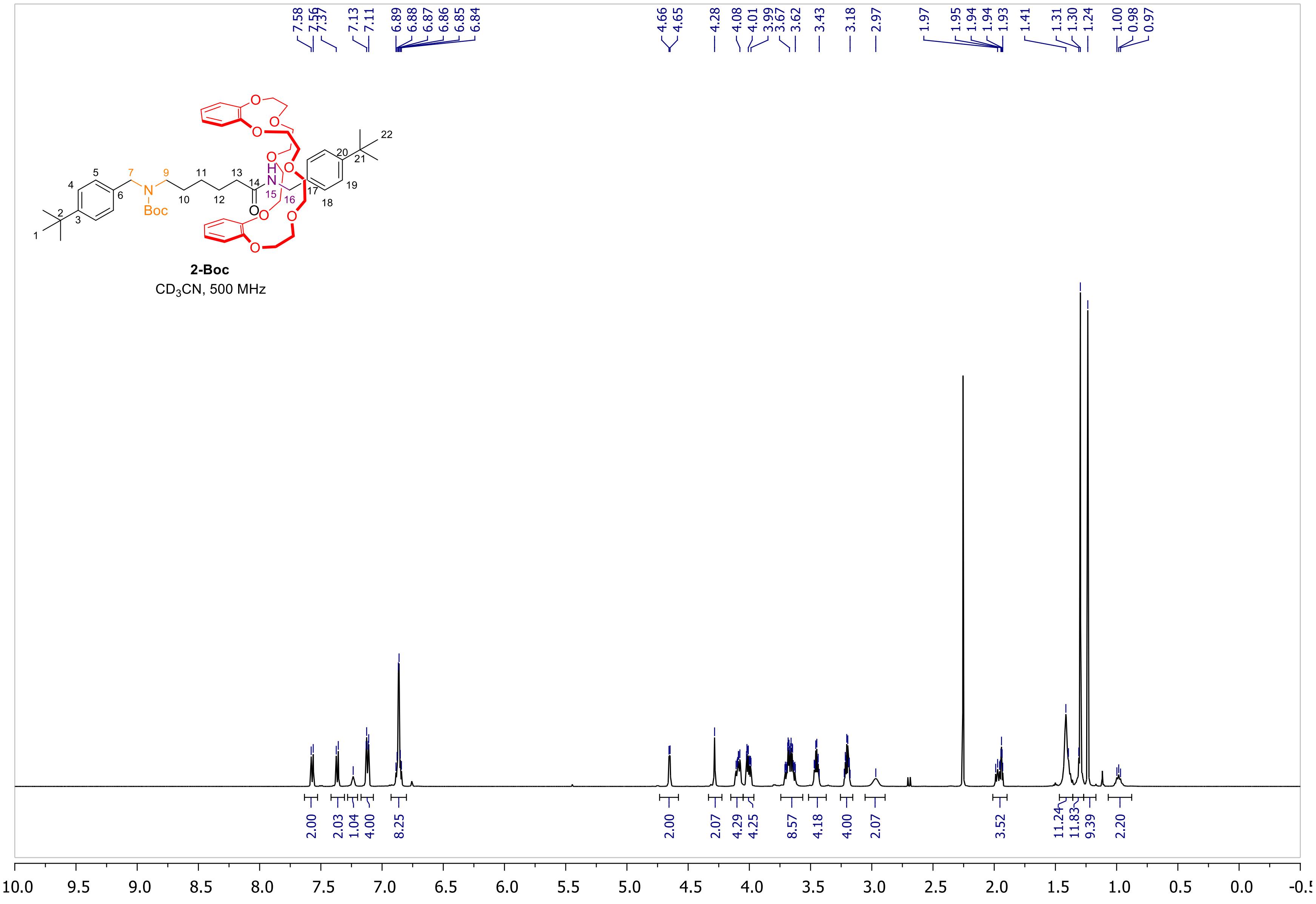
2-Boc
CD$_3$CN, 500 MHz
Boc

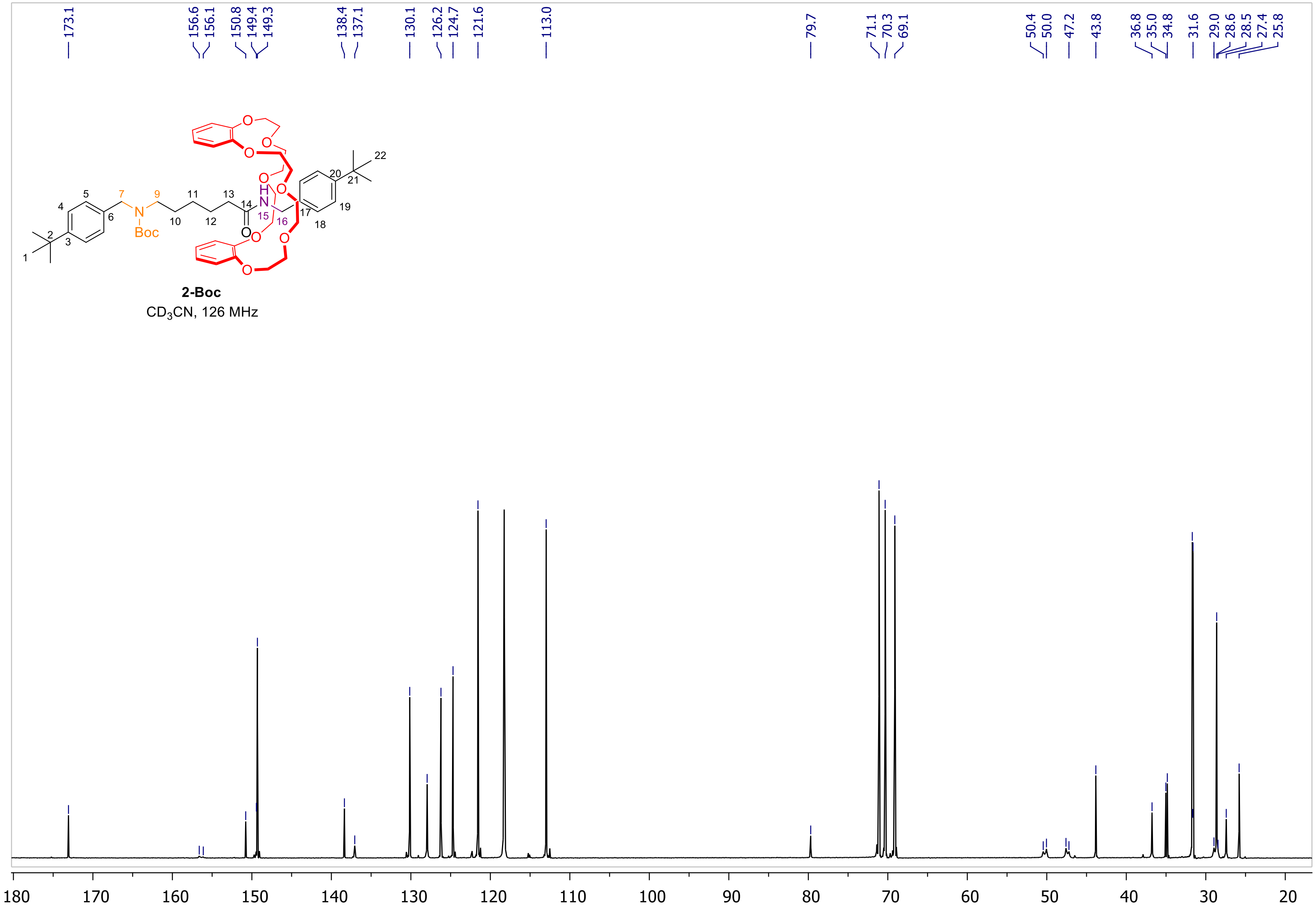
173.1
156.6
156.1
150.8
149.4
149.3
138.4
137.1
130.1
126.2
124.7
121.6
113.0
79.7
71.1
70.3
69.1
50.4
50.0
47.2
43.8
36.8
35.0
34.8
31.6
29.0
28.6
28.5
27.4
25.8
2-Boc
CD3CN, 126 MHz
180
170
160
150
140
130
120
110
100
90
80
70
60
50
40
30
20

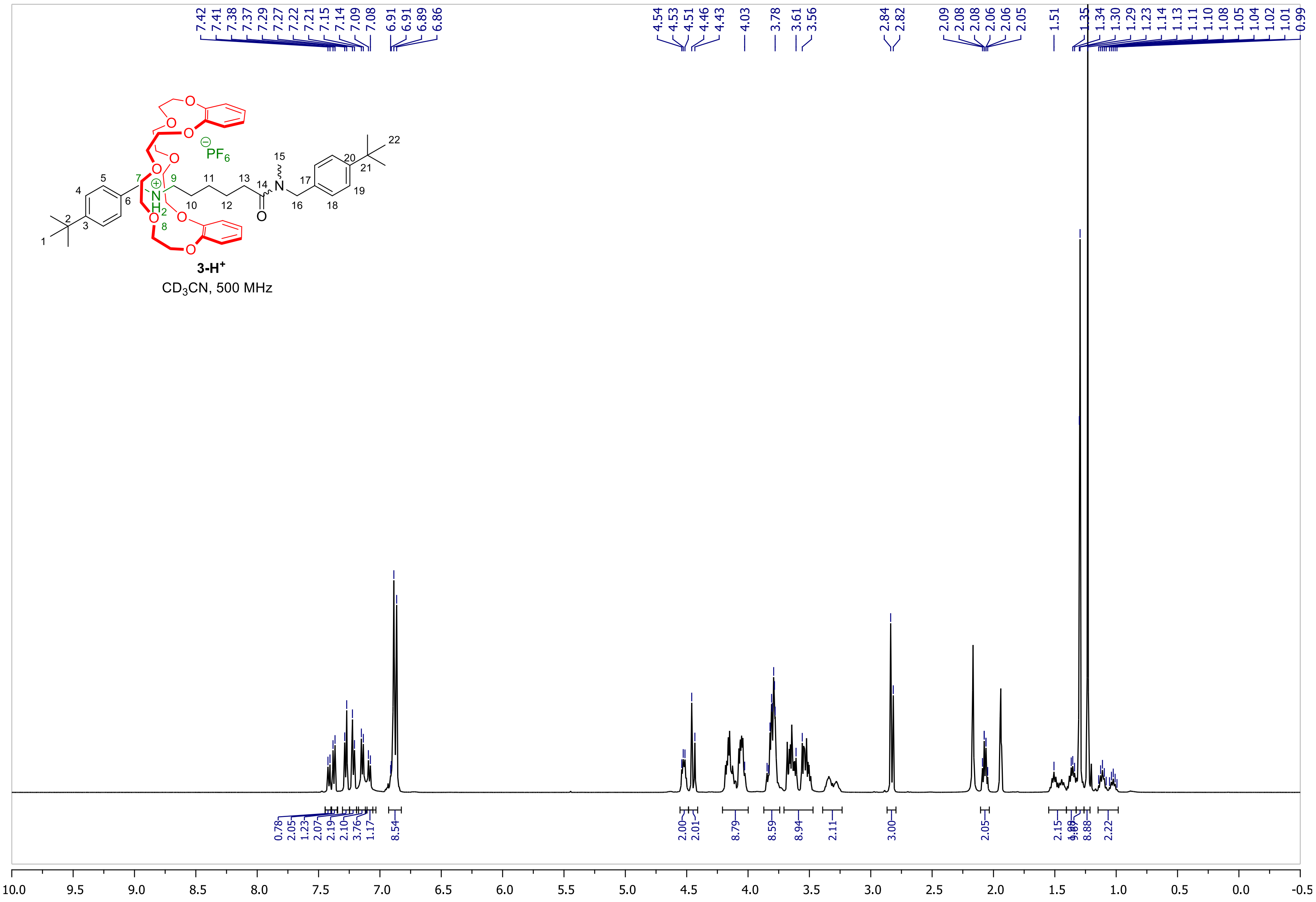
3-H+
CD3CN, 500 MHz

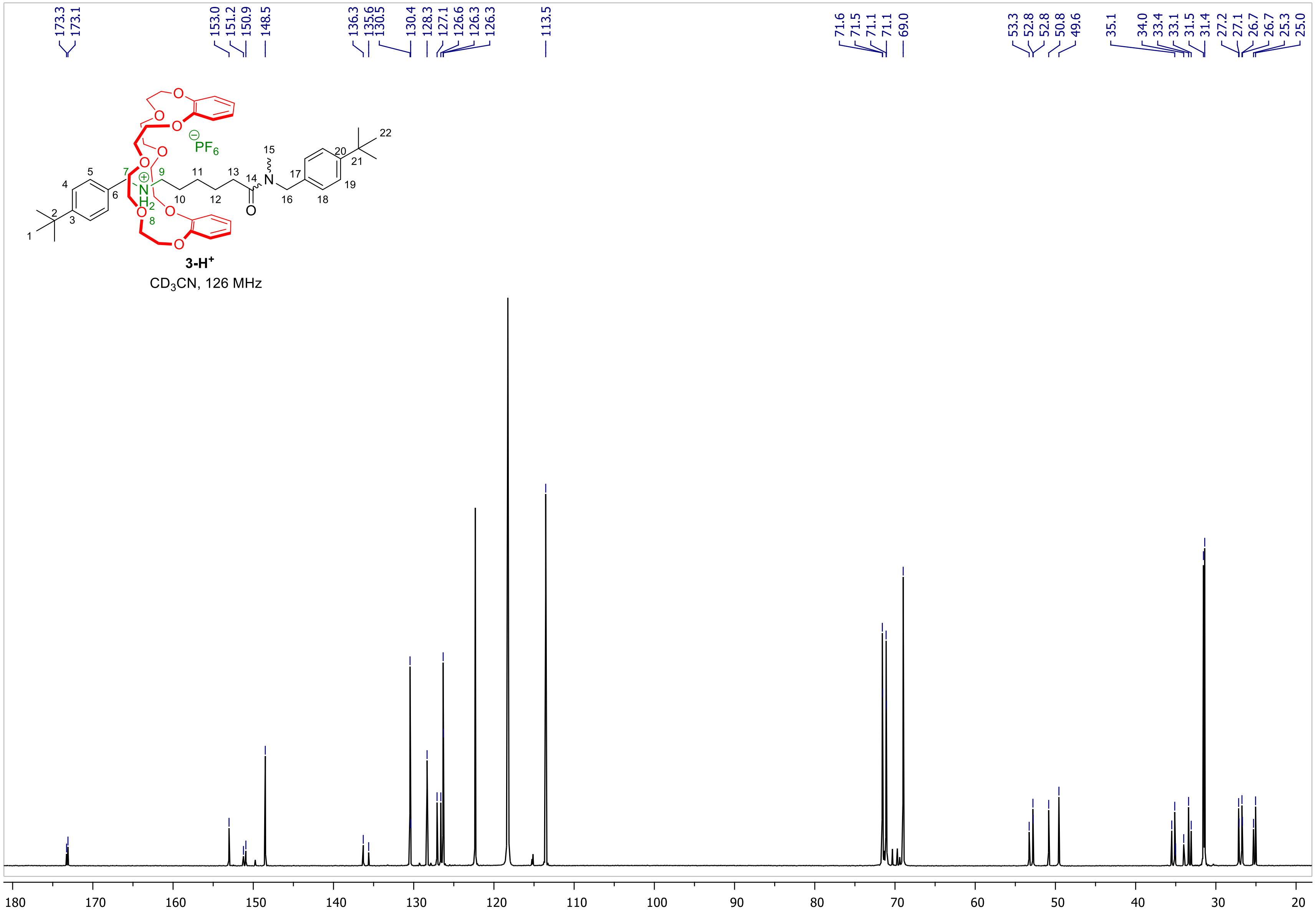
173.3
173.1
153.0
151.2
150.9
148.5
136.3
135.6
130.5
130.4
128.3
127.1
126.6
126.3
126.3
113.5
71.6
71.5
71.1
71.1
69.0
53.3
52.8
52.8
50.8
49.6
35.1
34.0
33.4
33.1
31.5
31.4
27.2
27.1
26.7
26.7
25.3
25.0
3-H+
CD3CN, 126 MHz

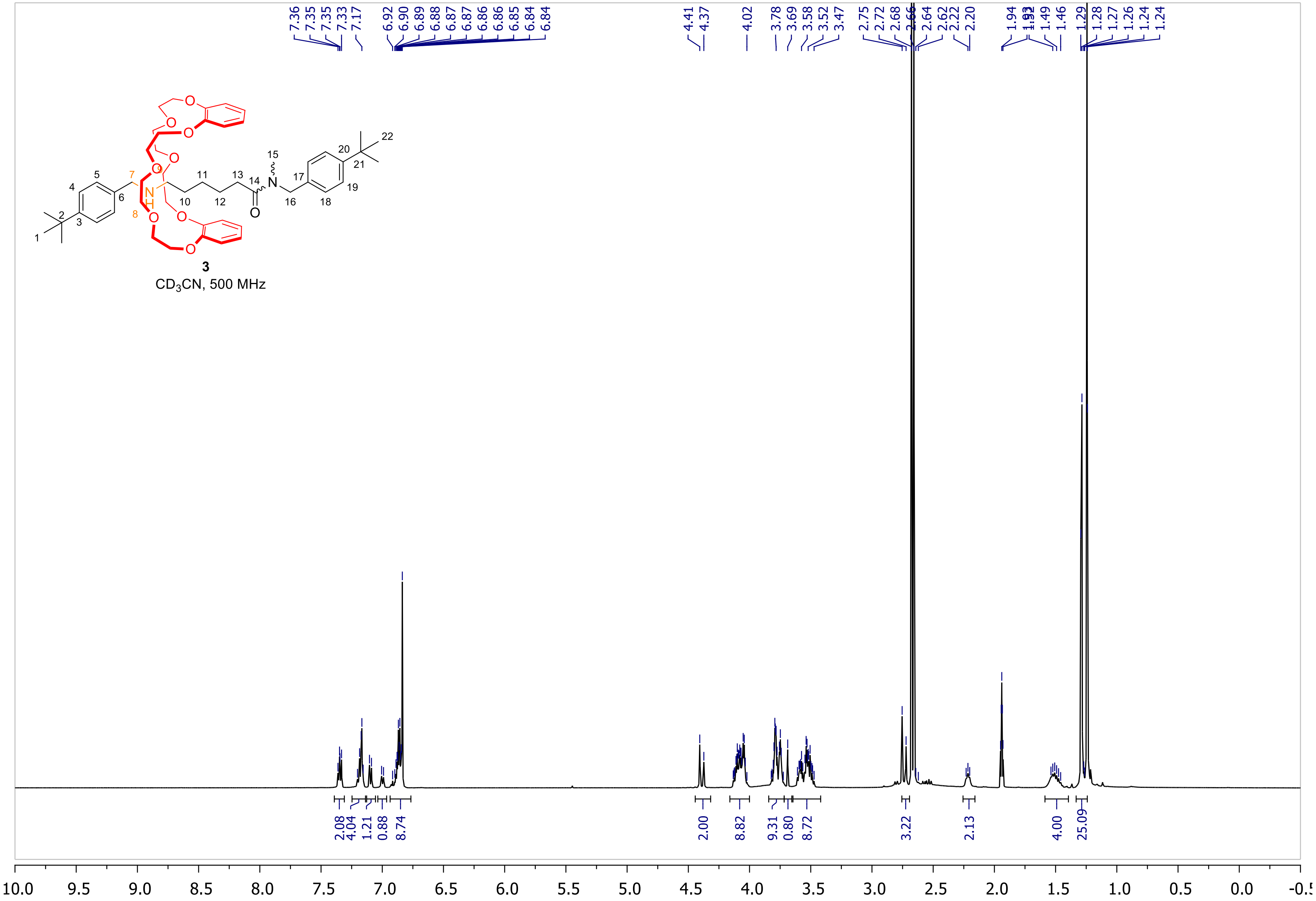
7.36 7.35 7.35 7.33 7.17 6.92 6.90 6.89 6.88 6.87 6.87 6.86 6.86 6.85 6.84 6.84
4.41 4.37 4.02 3.78 3.69 3.58 3.52 3.47 2.75 2.72 2.68 2.64 2.62 2.22 2.20 1.94 1.49 1.46 1.29 1.28 1.27 1.26 1.24 1.24
3
CD3CN, 500 MHz
2.08 4.04 1.21 0.88 8.74 2.00 8.82 9.31 0.80 8.72 3.22 2.13 4.00 25.09
10.0 9.5 9.0 8.5 8.0 7.5 7.0 6.5 6.0 5.5 5.0 4.5 4.0 3.5 3.0 2.5 2.0 1.5 1.0 0.5 0.0

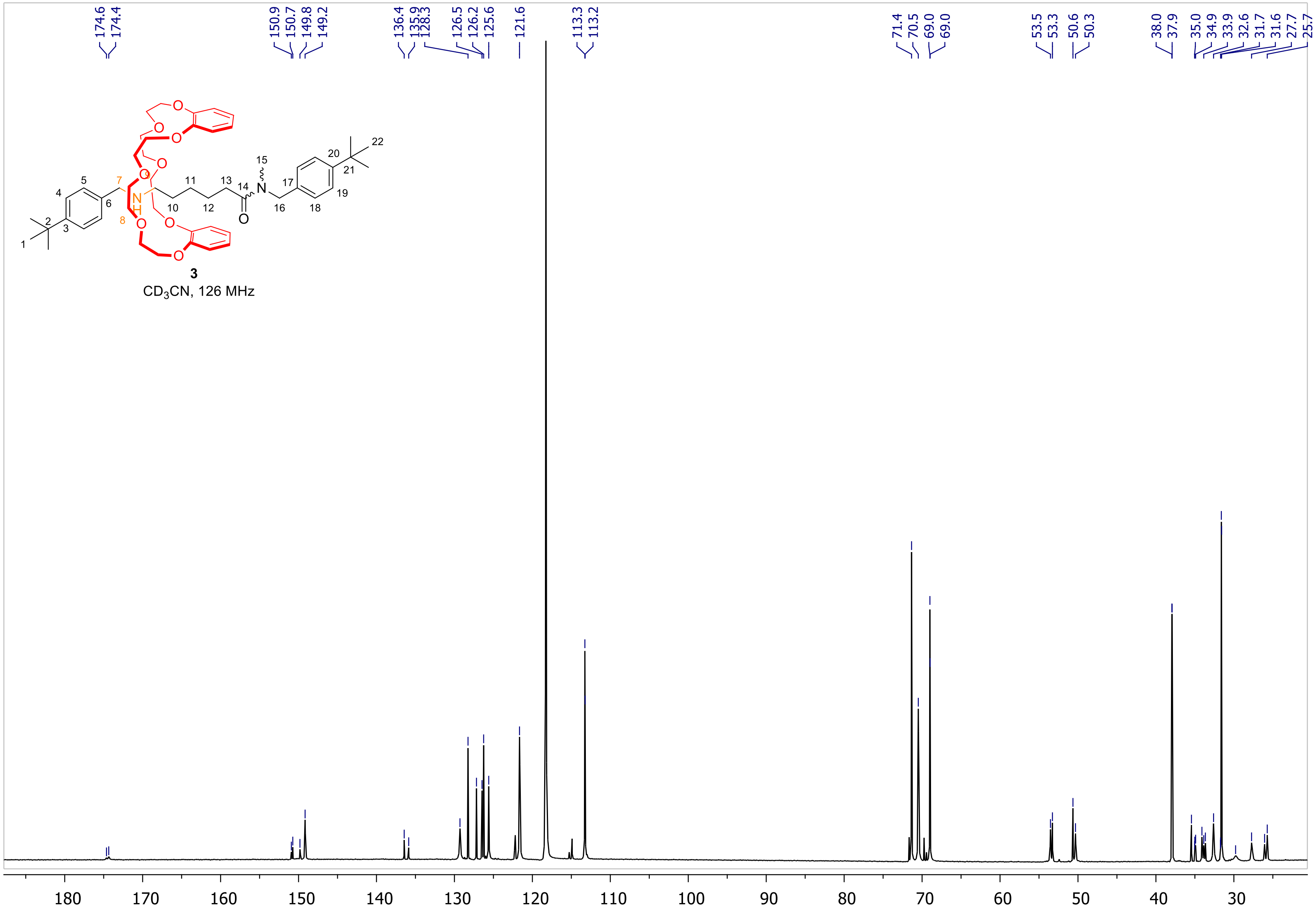
174.6
174.4
150.9
150.7
149.8
149.2
136.4
135.9
128.3
126.5
126.2
125.6
121.6
113.3
113.2
71.4
70.5
69.0
69.0
53.5
53.3
50.6
50.3
38.0
37.9
35.0
34.9
33.9
32.6
31.7
31.6
27.7
25.7
3
CD3CN, 126 MHz
180
170
160
150
140
130
120
110
100
90
80
70
60
50
40
30

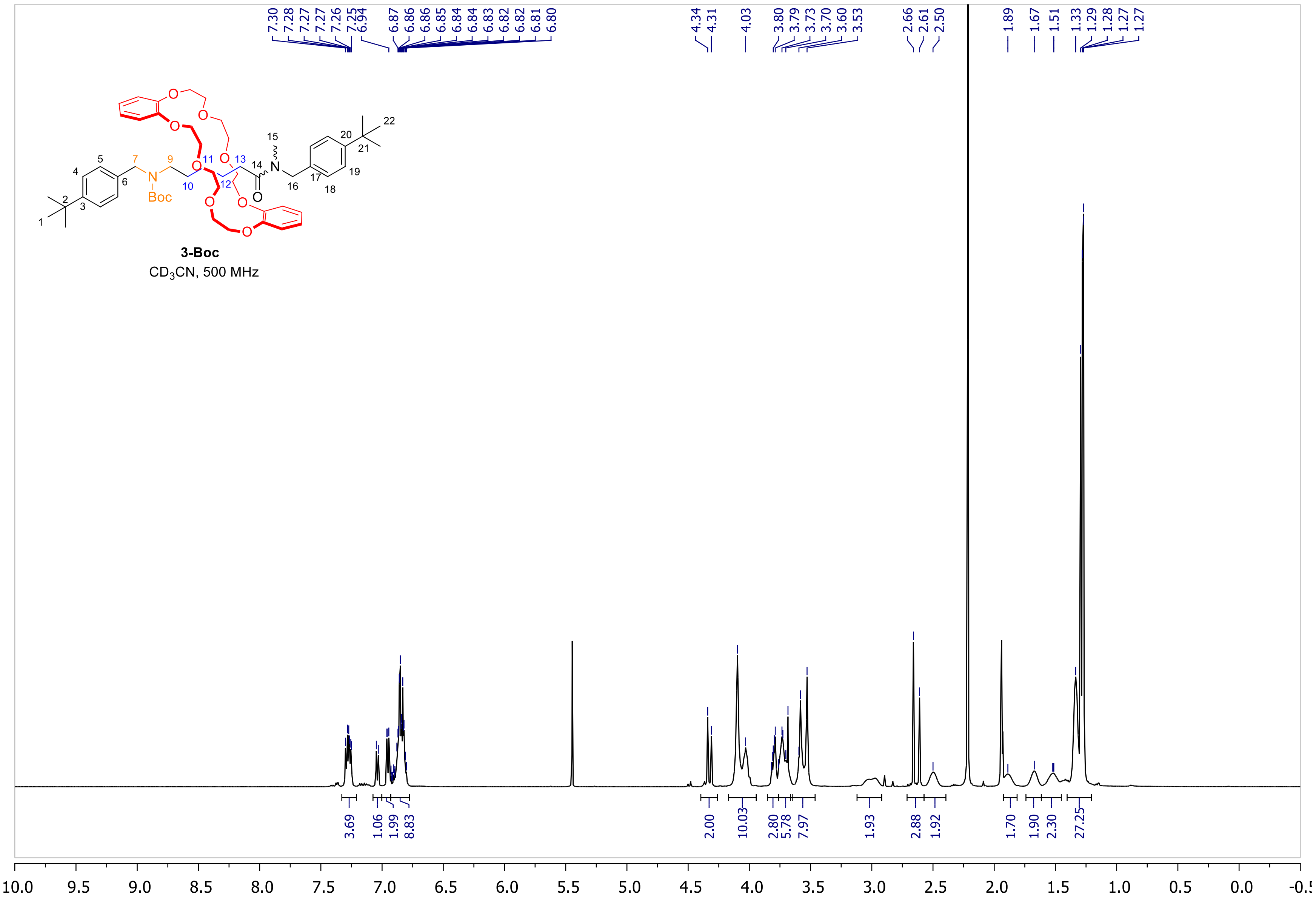
3-Boc
CD3CN, 500 MHz

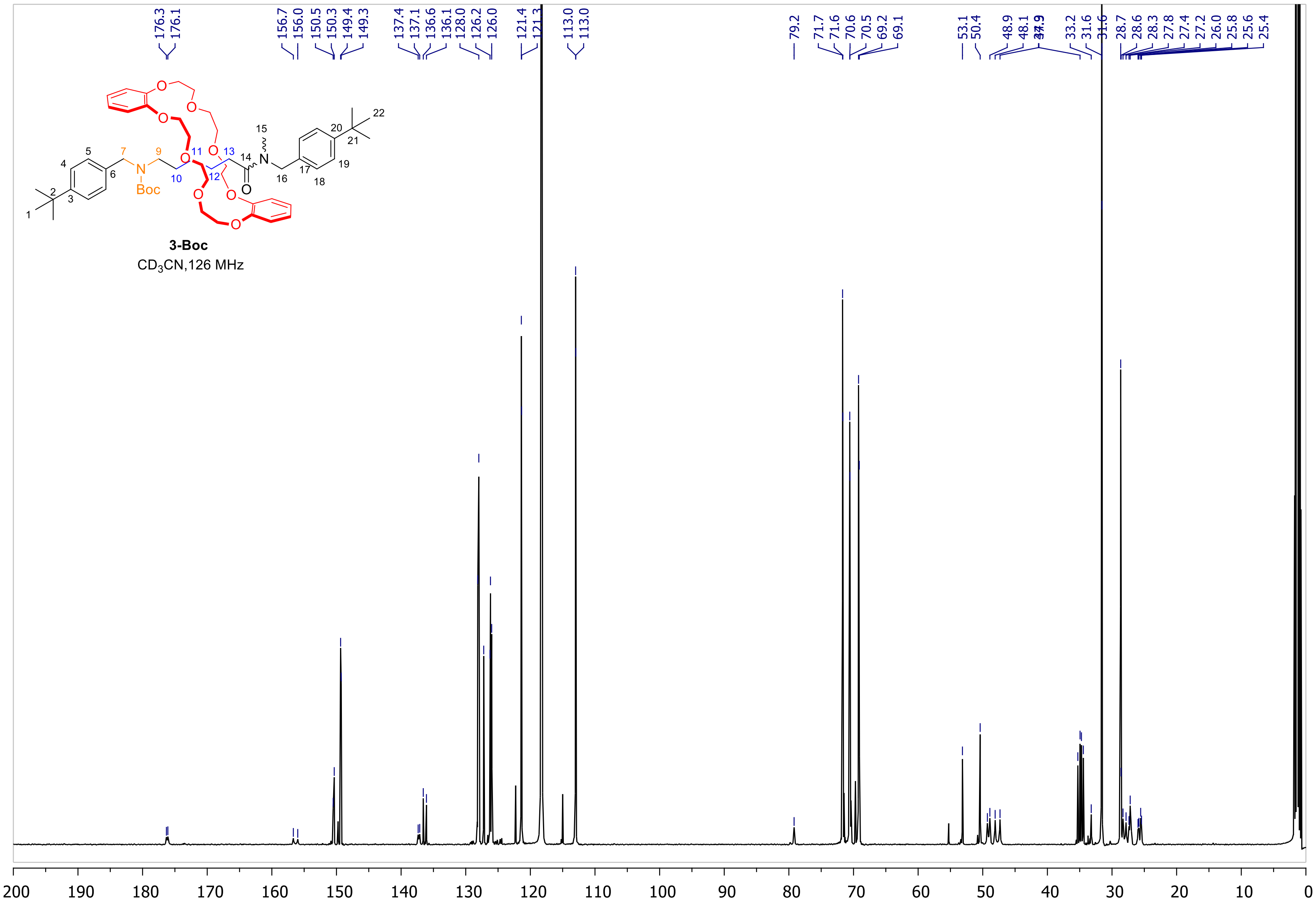

3-Boc
CD_3CN,126 MHz
176.3
176.1
156.7
156.0
150.5
150.3
149.4
149.3
137.4
137.1
136.6
136.1
128.0
126.2
126.0
121.4
113.0
113.0
79.2
71.7
71.6
70.6
70.5
69.2
69.1
53.1
50.4
48.9
48.1
33.2
31.6
28.7
28.6
28.3
27.8
27.4
27.2
26.0
25.8
25.6
25.4

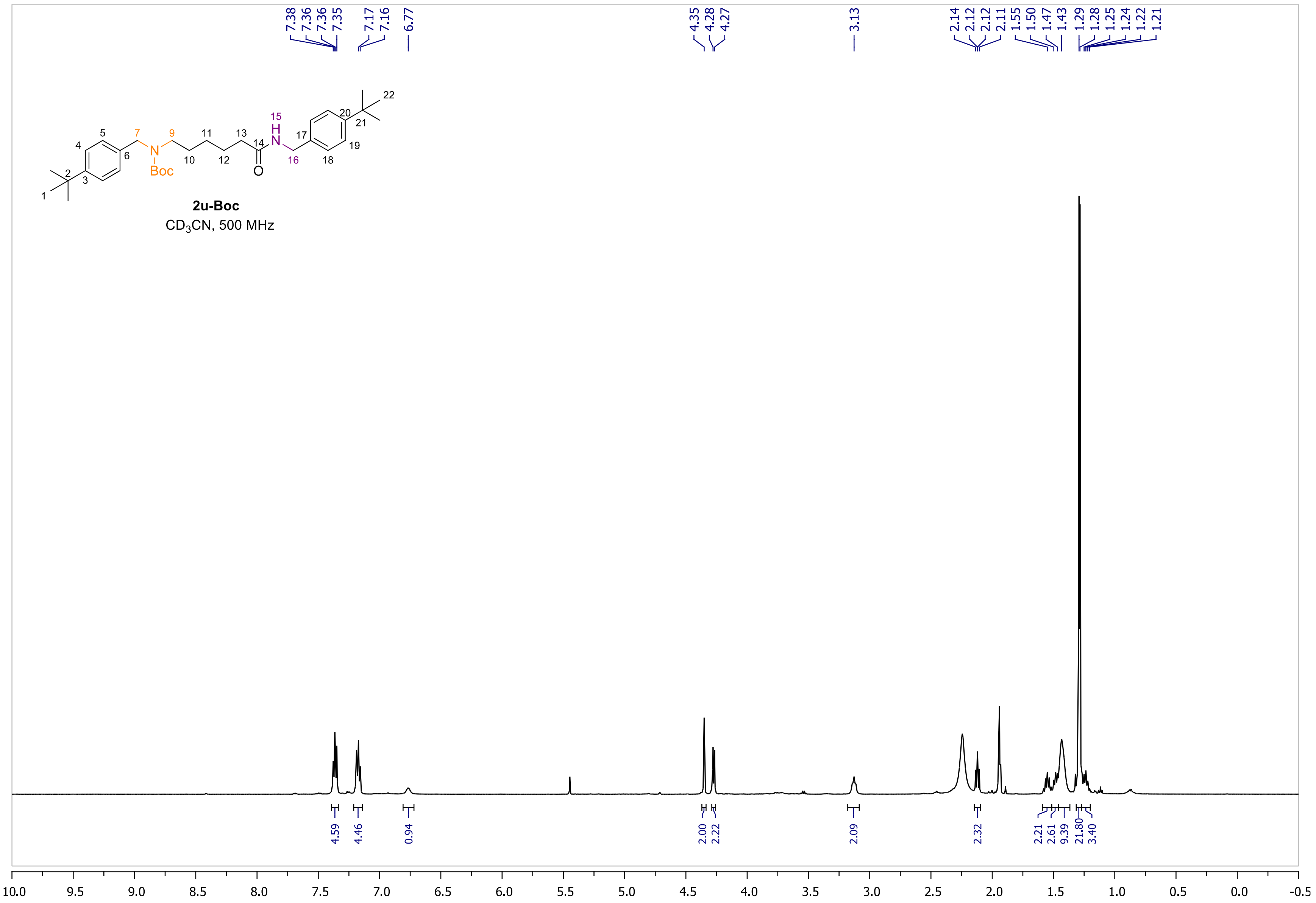
7.38
7.36
7.36
7.35
7.17
7.16
6.77
4.35
4.28
4.27
3.13
2.14
2.12
2.12
2.11
1.55
1.50
1.47
1.43
1.29
1.28
1.25
1.24
1.22
1.21
Boc
2u-Boc
CD3CN, 500 MHz
4.59
4.46
0.94
2.00
2.22
2.09
2.32
2.21
2.61
9.39
21.80
3.40
10.0 9.5 9.0 8.5 8.0 7.5 7.0 6.5 6.0 5.5 5.0 4.5 4.0 3.5 3.0 2.5 2.0 1.5 1.0 0.5 0.0 -0.5

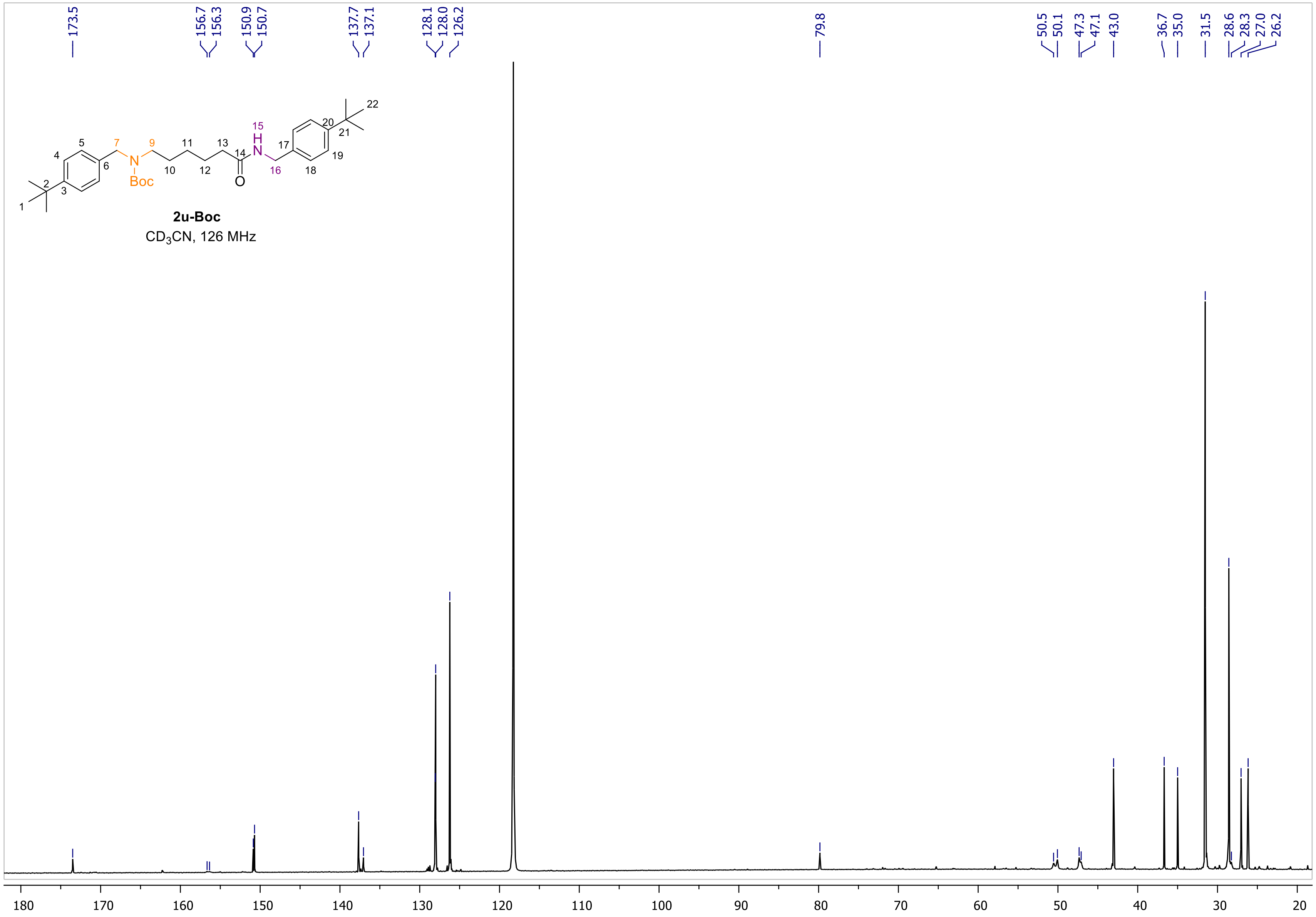

173.5
156.7
156.3
150.9
150.7
137.7
137.1
128.1
128.0
126.2
79.8
50.5
50.1
47.3
47.1
43.0
36.7
35.0
31.5
28.6
28.3
27.0
26.2
Boc
2u-Boc
CD3CN, 126 MHz
180 170 160 150 140 130 120 110 100 90 80 70 60 50 40 30 20

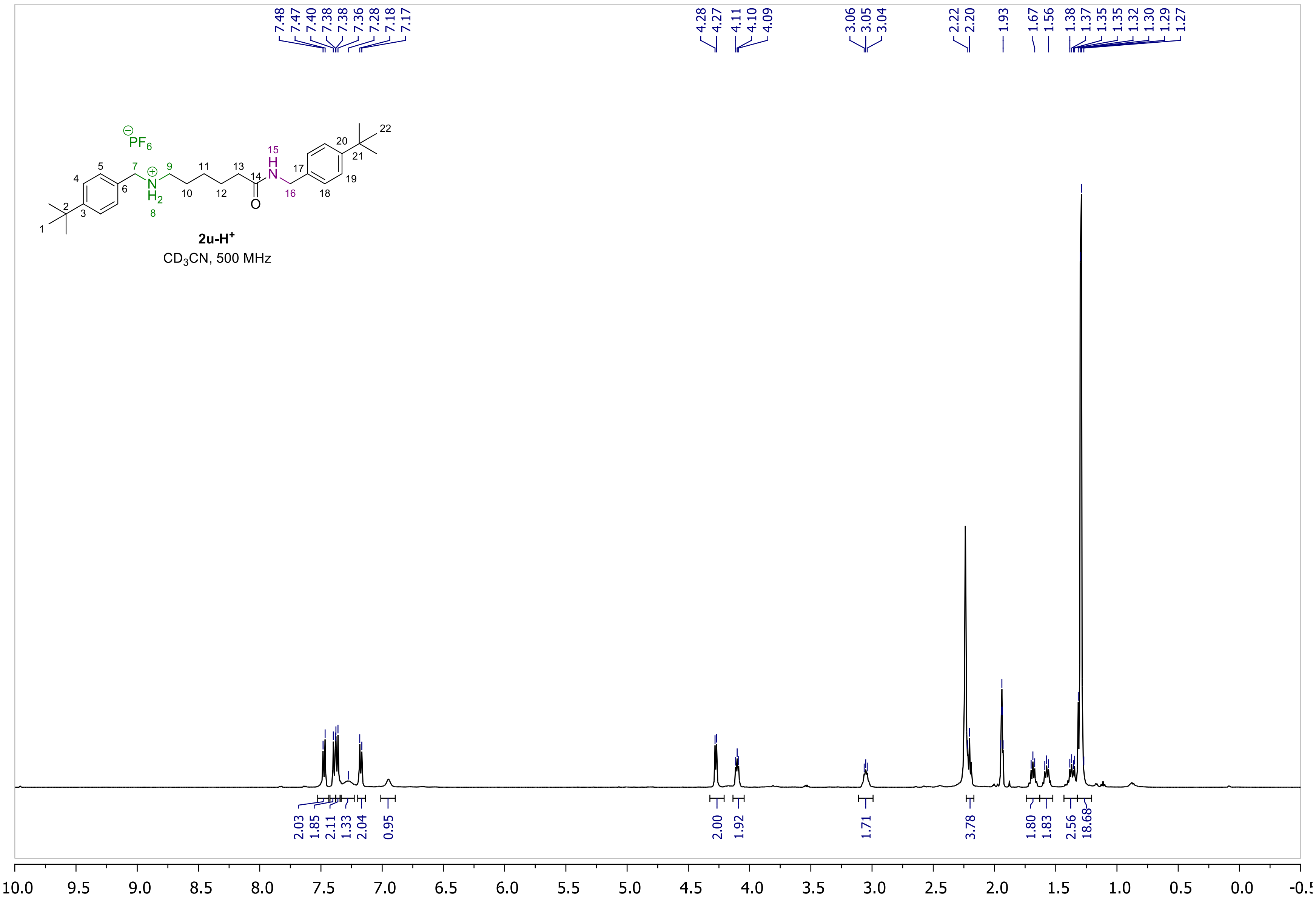
7.48 7.47 7.40 7.38 7.38 7.36 7.28 7.18 7.17
4.28 4.27 4.11 4.10 4.09
3.06 3.05 3.04
2.22 2.20
1.93
1.67 1.56
1.38 1.37 1.35 1.35 1.32 1.30 1.29 1.27
PF6
2u-H+
CD3CN, 500 MHz
2.03 1.85 2.11 1.33 2.04 0.95
2.00 1.92
1.71
3.78
1.80 1.83 2.56 18.68
10.0 9.5 9.0 8.5 8.0 7.5 7.0 6.5 6.0 5.5 5.0 4.5 4.0 3.5 3.0 2.5 2.0 1.5 1.0 0.5 0.0

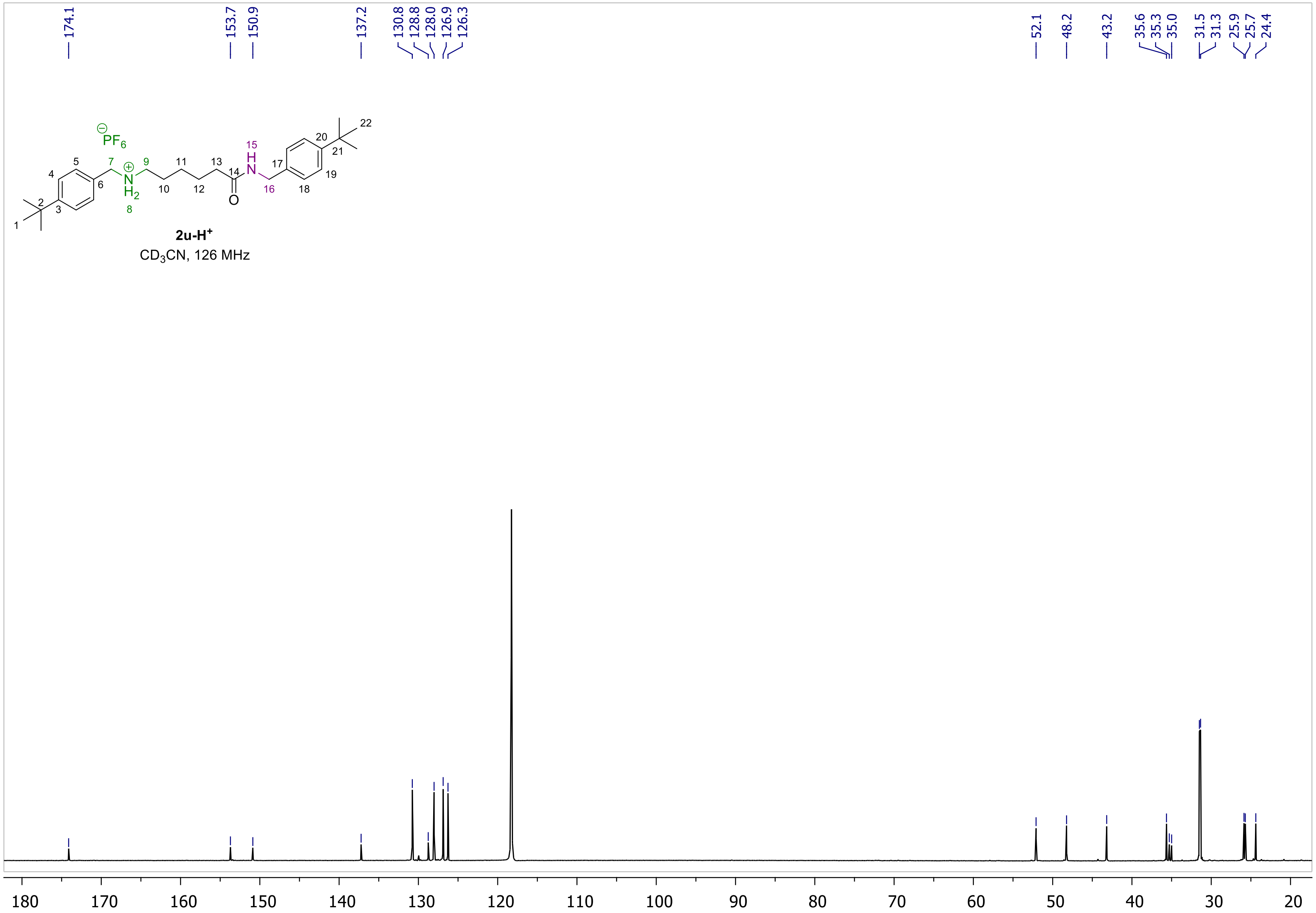
174.1
153.7
150.9
137.2
130.8
128.8
128.0
126.9
126.3
52.1
48.2
43.2
35.6
35.3
35.0
31.5
31.3
25.9
25.7
24.4
2u-H+
CD3CN, 126 MHz

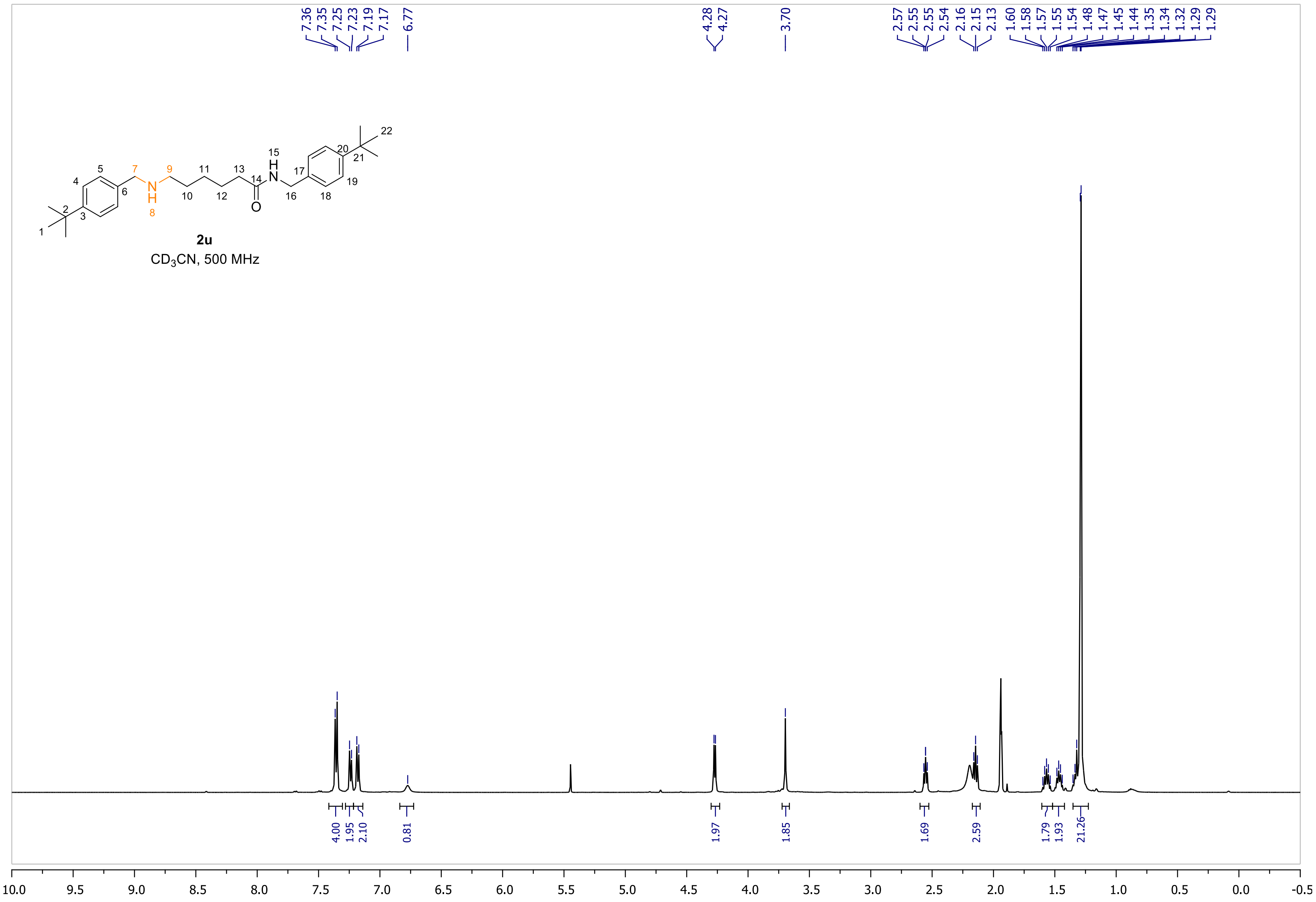

2u

CD_3CN, 500 MHz

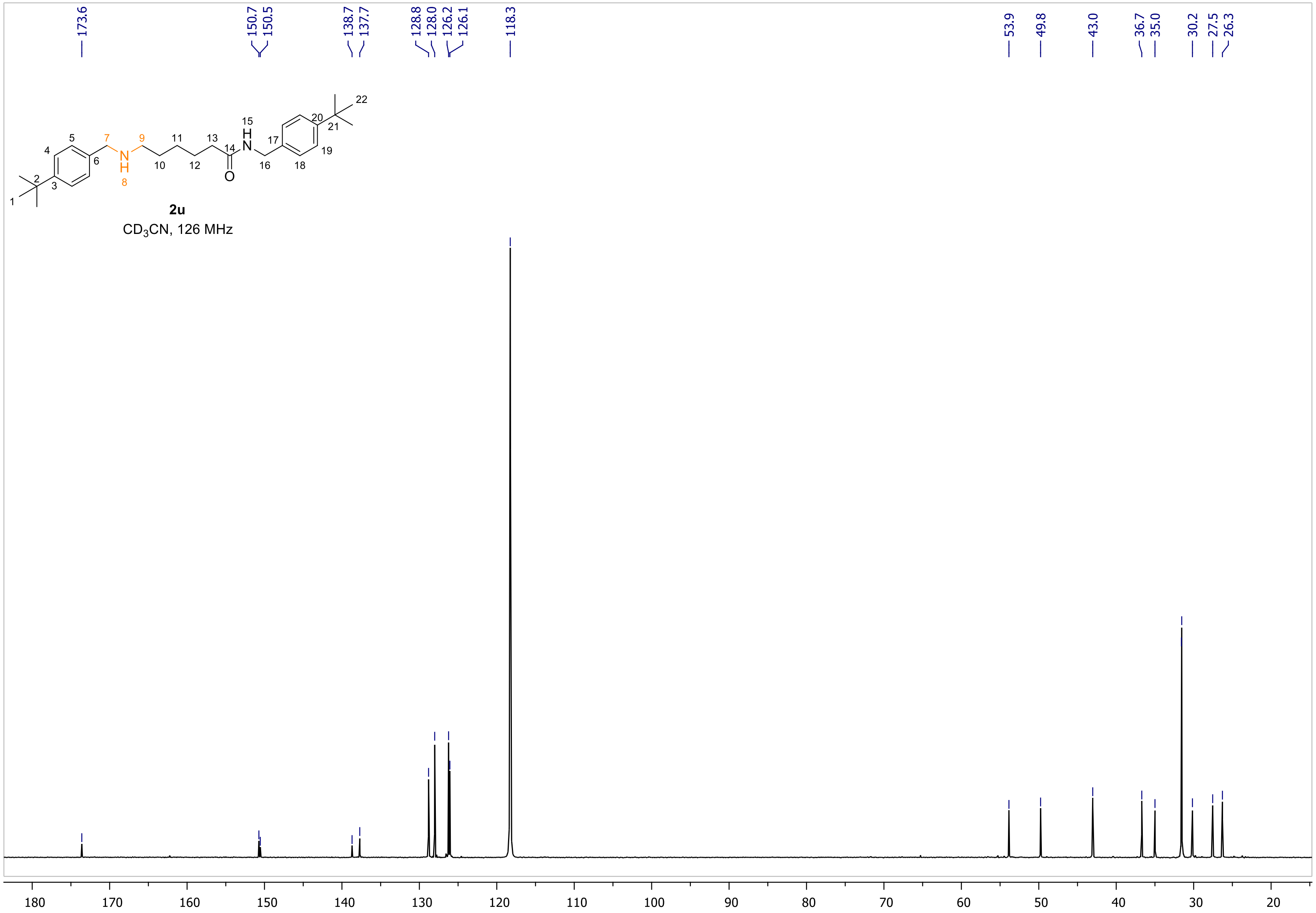
173.6
150.7
150.5
138.7
137.7
128.8
128.0
126.2
126.1
118.3
53.9
49.8
43.0
36.7
35.0
30.2
27.5
26.3
2u
CD3CN, 126 MHz
180 170 160 150 140 130 120 110 100 90 80 70 60 50 40 30 20

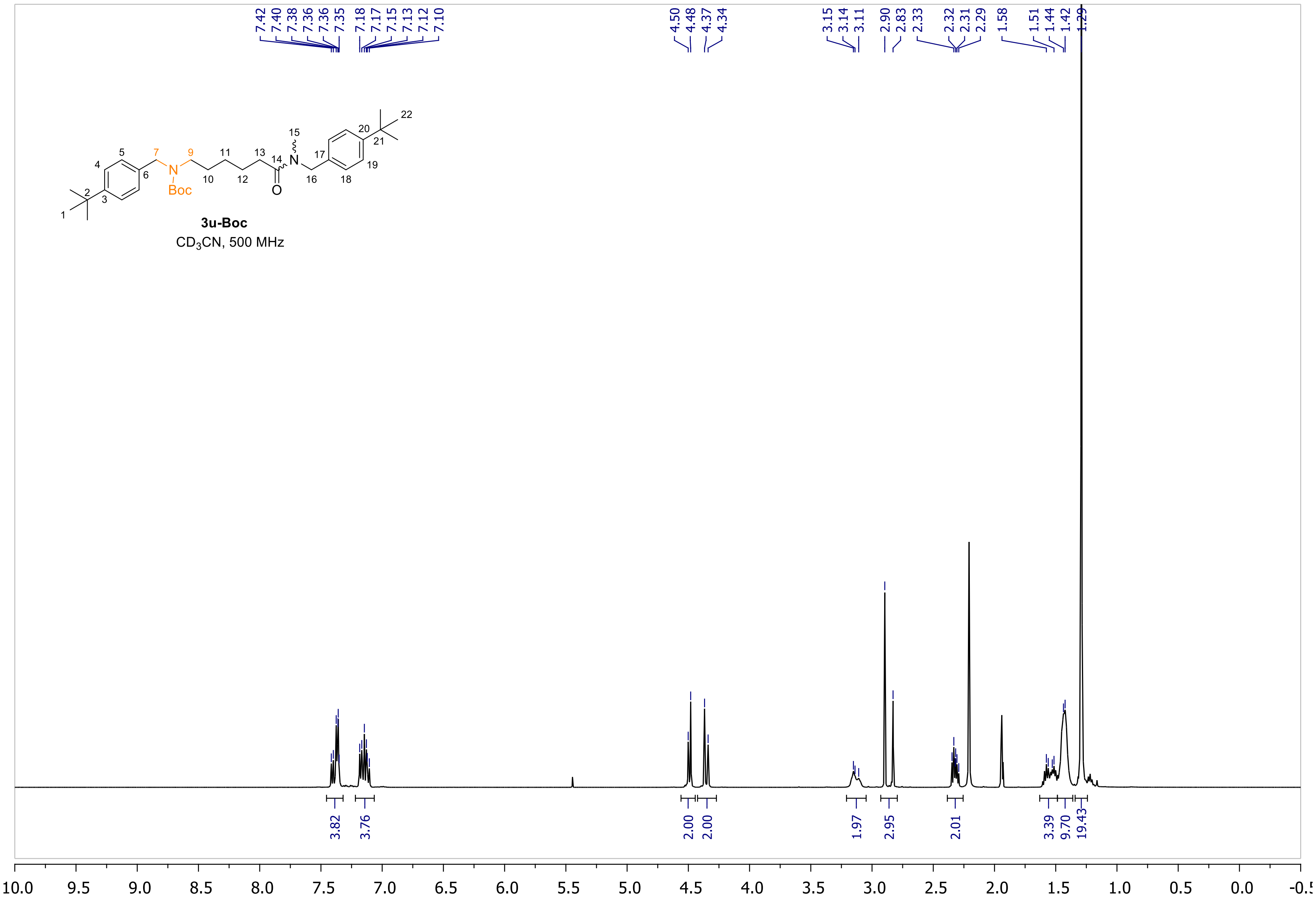
3u-Boc
CD3CN, 500 MHz
Boc
7.42 7.40 7.38 7.36 7.36 7.35 7.18 7.17 7.15 7.13 7.12 7.10
4.50 4.48 4.37 4.34
3.15 3.14 3.11 2.90 2.83 2.33 2.32 2.31 2.29 1.58 1.51 1.44 1.42 1.29
3.82 3.76 2.00 2.00 1.97 2.95 2.01 3.39 9.70 19.43
10.0 9.5 9.0 8.5 8.0 7.5 7.0 6.5 6.0 5.5 5.0 4.5 4.0 3.5 3.0 2.5 2.0 1.5 1.0 0.5 0.0

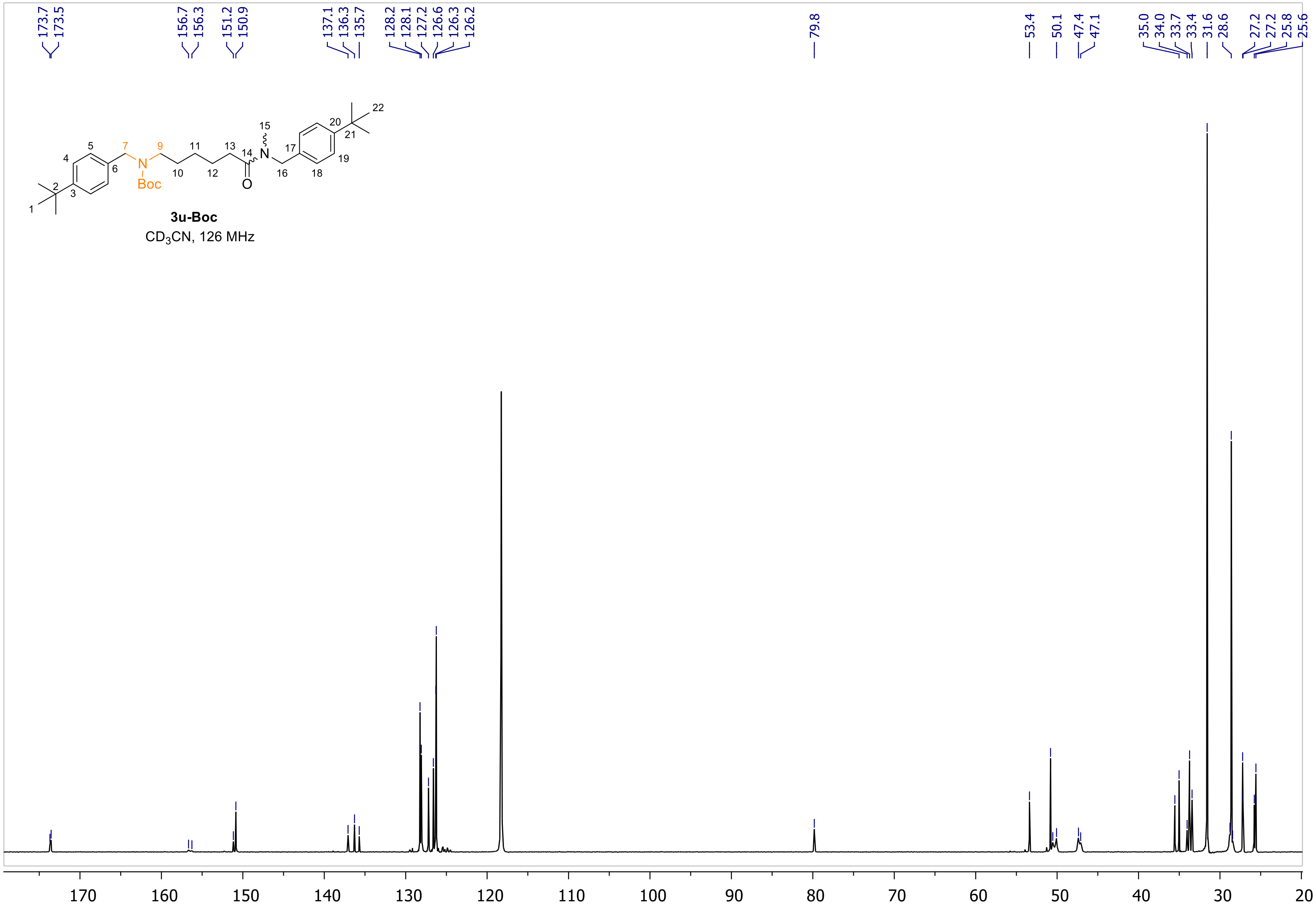
173.7
173.5
156.7
156.3
151.2
150.9
137.1
136.3
135.7
128.2
128.1
127.2
126.6
126.3
126.2
79.8
53.4
50.1
47.4
47.1
35.0
34.0
33.7
33.4
31.6
28.6
27.2
27.2
25.8
25.6
Boc
3u-Boc
CD3CN, 126 MHz
170
160
150
140
130
120
110
100
90
80
70
60
50
40
30
20

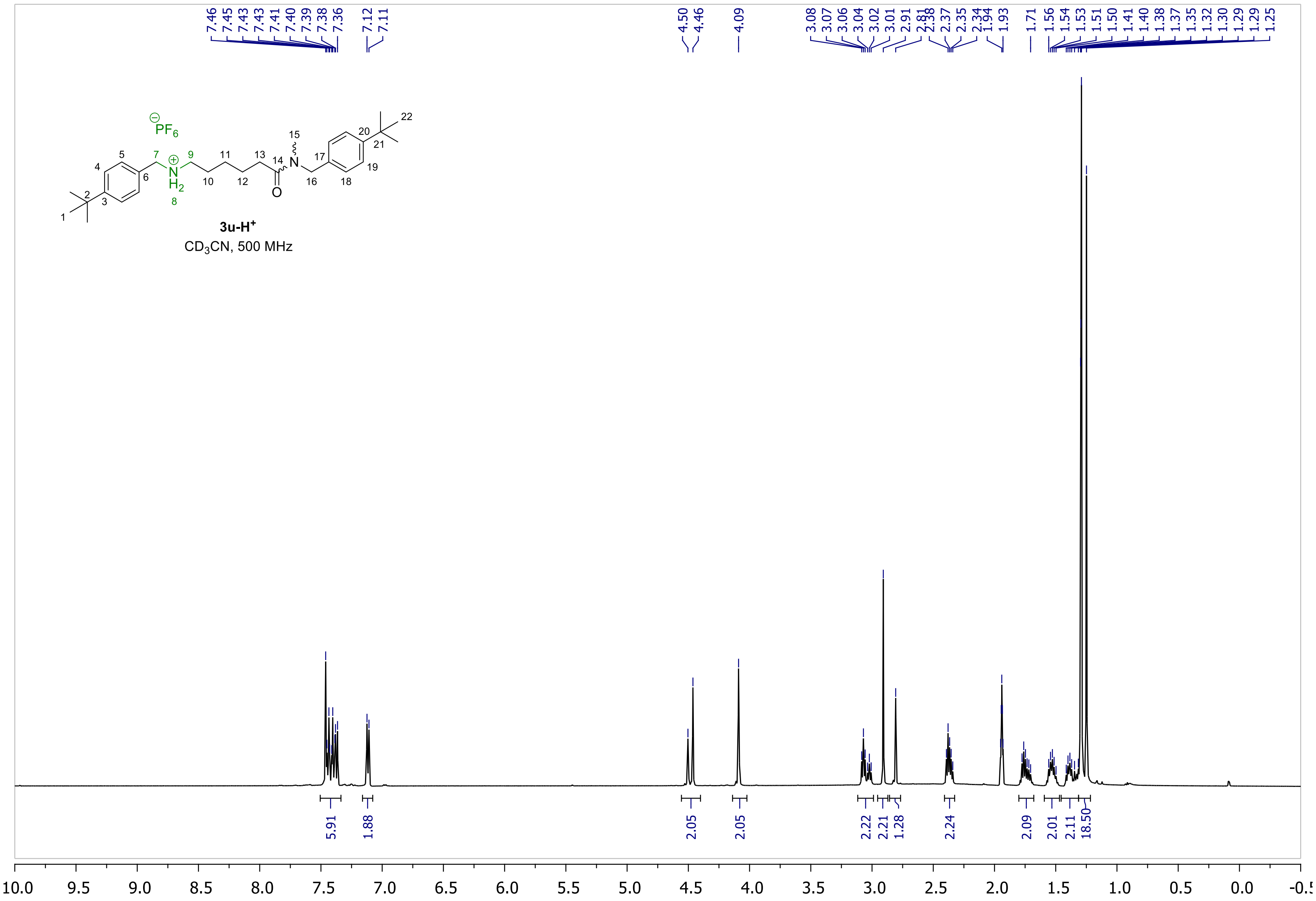
3u-H+
CD3CN, 500 MHz

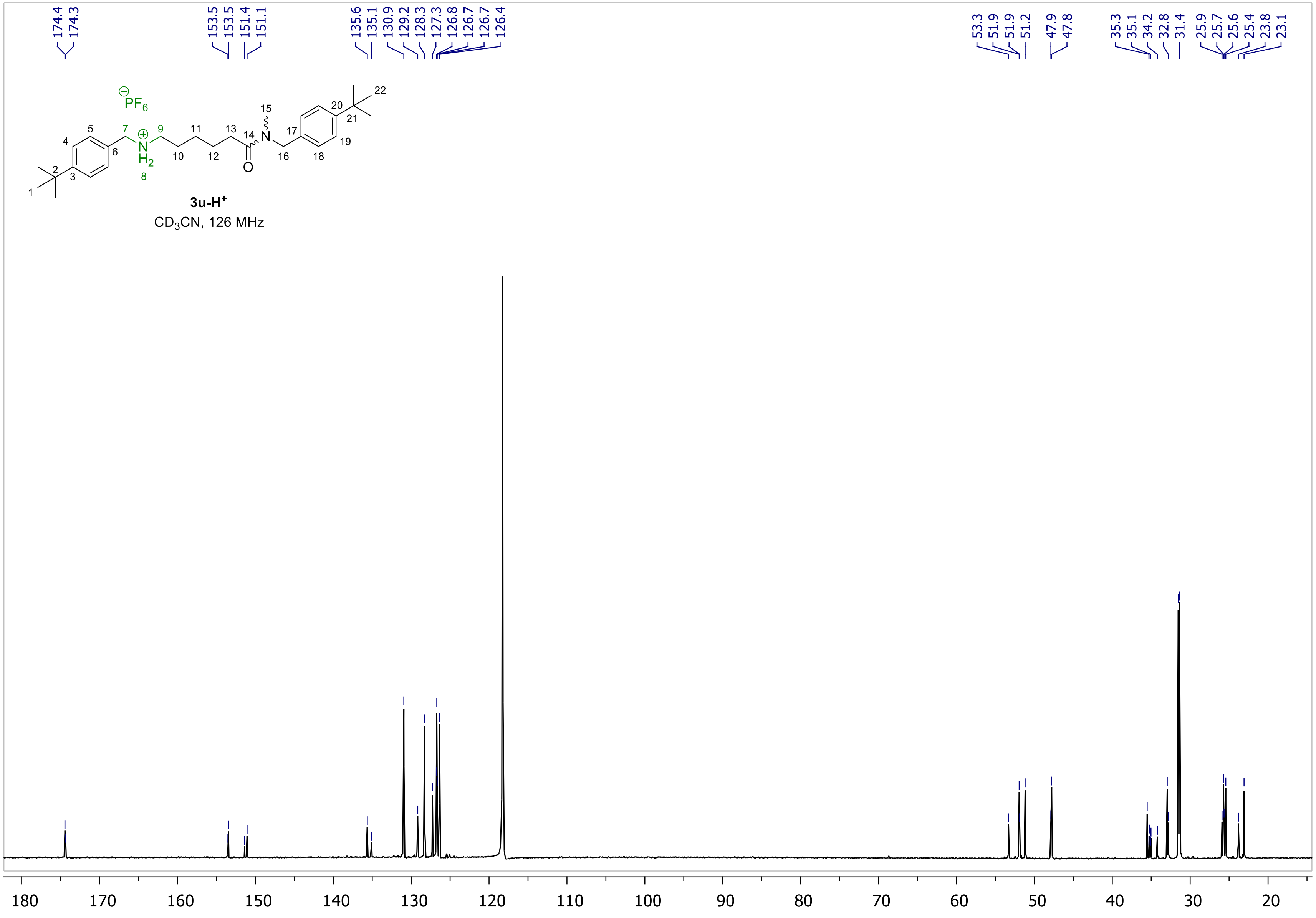
174.4
174.3
153.5
153.5
151.4
151.1
135.6
135.1
130.9
129.2
128.3
127.3
126.8
126.7
126.7
126.4
53.3
51.9
51.9
51.2
47.9
47.8
35.3
35.1
34.2
32.8
31.4
25.9
25.7
25.6
25.4
23.8
23.1
3u-H+
CD3CN, 126 MHz
180 170 160 150 140 130 120 110 100 90 80 70 60 50 40 30 20

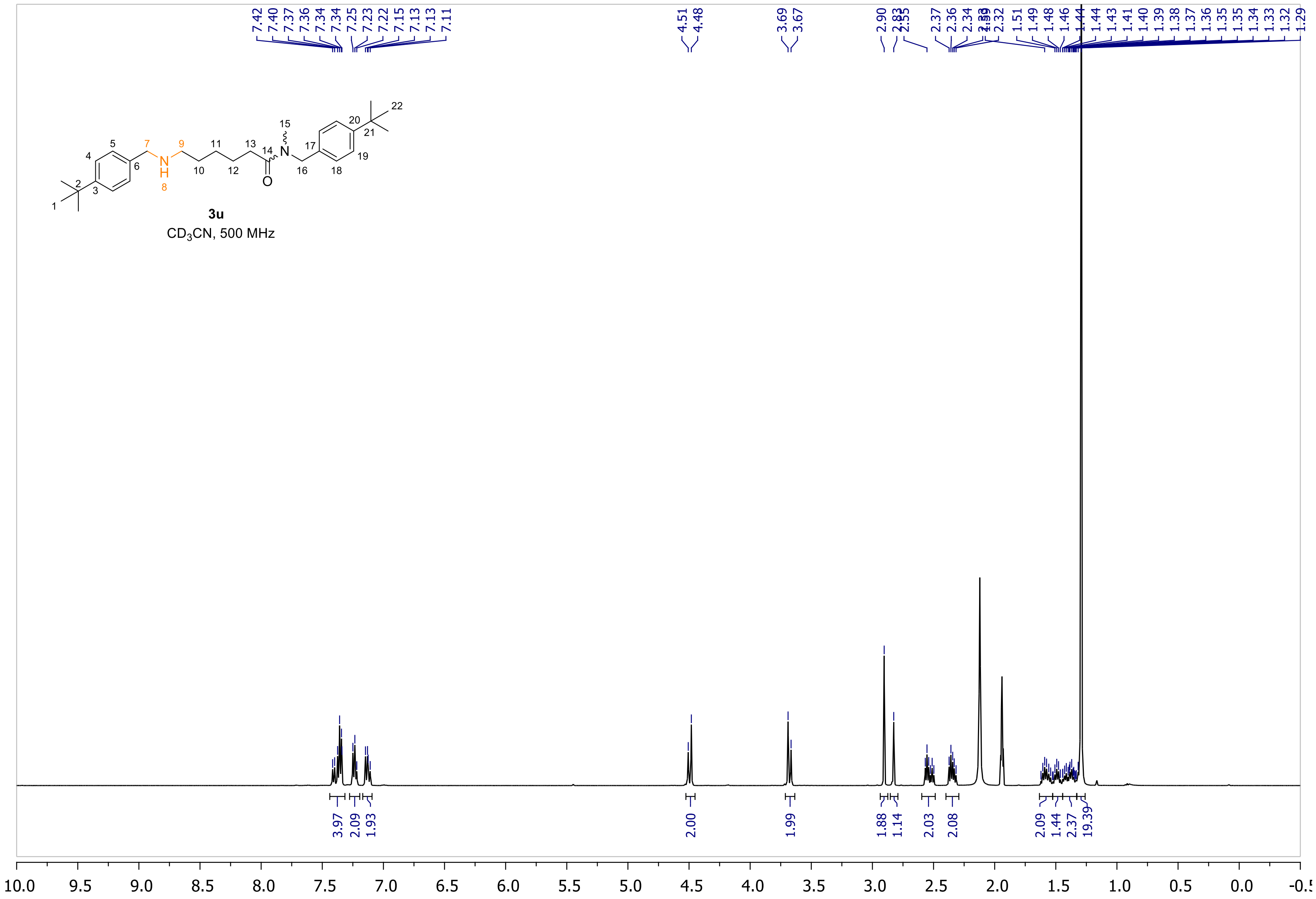
3u
CD$_3$CN, 500 MHz

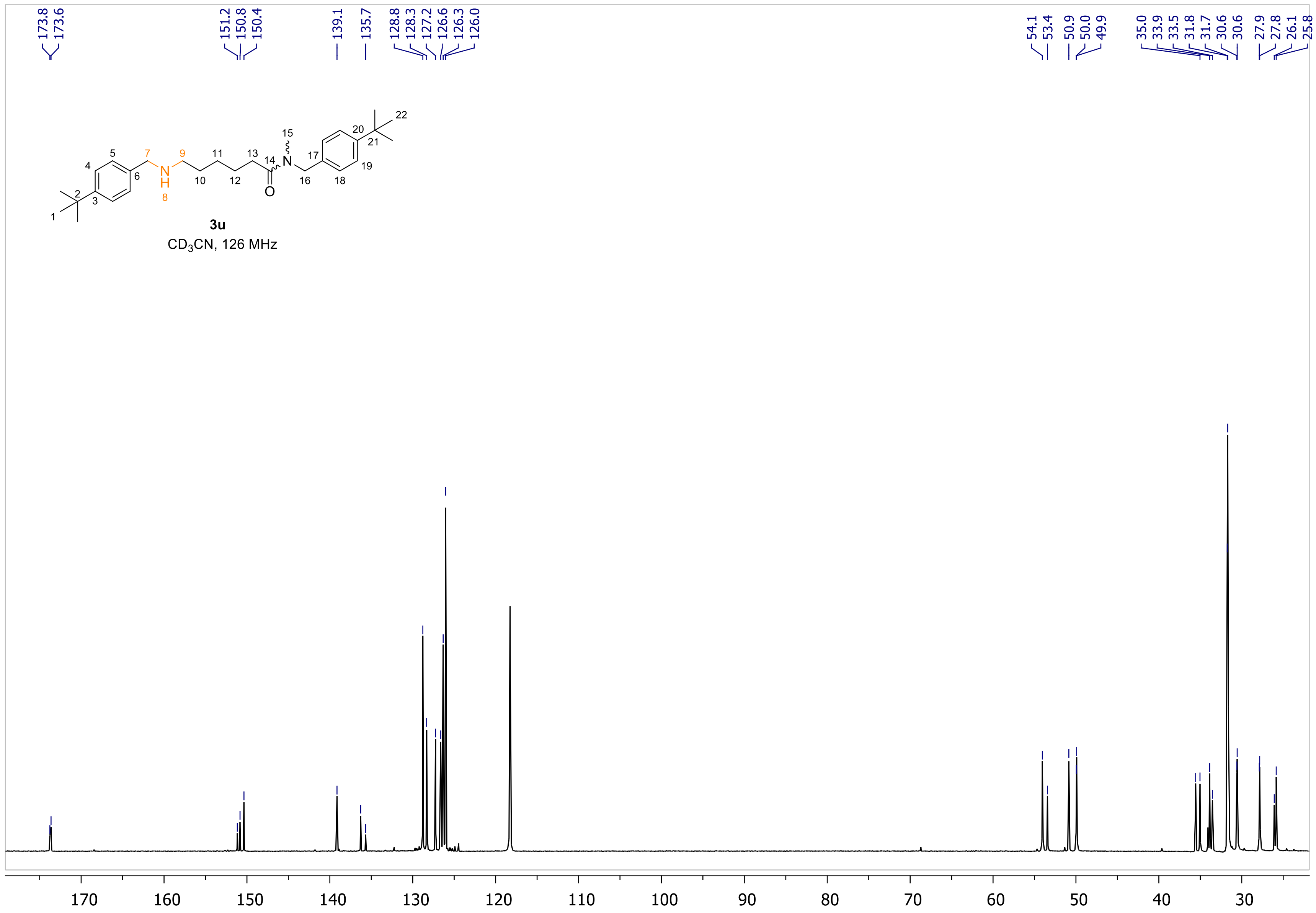

173.8
173.6
151.2
150.8
150.4
139.1
135.7
128.8
128.3
127.2
126.6
126.3
126.0
54.1
53.4
50.9
50.0
49.9
35.0
33.9
33.5
31.8
31.7
30.6
30.6
27.9
27.8
26.1
25.8
3u
CD3CN, 126 MHz
170
160
150
140
130
120
110
100
90
80
70
60
50
40
30
